# Dominant inhibition of awn development by a putative zinc‐finger transcriptional repressor expressed at the *B1* locus in wheat

**DOI:** 10.1111/nph.16154

**Published:** 2019-10-10

**Authors:** Daiqing Huang, Qian Zheng, Tancey Melchkart, Yasmina Bekkaoui, David J. F. Konkin, Sateesh Kagale, Martial Martucci, Frank M. You, Martha Clarke, Nikolai M. Adamski, Catherine Chinoy, Andrew Steed, Curt A. McCartney, Adrian J. Cutler, Paul Nicholson, J. Allan Feurtado

**Affiliations:** ^1^ Aquatic and Crop Resource Development National Research Council of Canada Saskatoon SK S7N 0W9 Canada; ^2^ Morden Research and Development Centre Agriculture and Agri‐Food Canada 101 Route 100 Morden MB R6M 1Y5 Canada; ^3^ Ottawa Research and Development Centre Agriculture and Agri‐Food Canada 960 Carling Avenue Ottawa ON K1A 0C6 Canada; ^4^ Department of Crop Genetics John Innes Centre Norwich Research Park, Colney Lane Norwich NR4 7UH UK

**Keywords:** awn, awnletted, *B1* locus, C2H2, EAR motif, transcriptional repression, wheat, zinc finger

## Abstract

- Awns, bristle‐like structures extending from grass lemmas, provide protection against predators, contribute to photosynthesis and aid in grain dispersal. In wheat, selection of awns with minimal extension, termed awnletted, has occurred during domestication by way of loci that dominantly inhibit awn development, such as *Tipped1 (B1)*,* Tipped2 (B2)*, and *Hooded (Hd)*. Here we identify and characterize the *B1* gene.
- *B1* was identified using bulked segregant RNA‐sequencing of an F_2_ durum wheat population and through deletion mapping of awned bread wheat mutants. Functional characterization was accomplished by gene overexpression while haplotype analyses assessed *B1* polymorphisms and genetic variation.
- Located on chromosome 5A, *B1* is a C2H2 zinc finger encoding gene with ethylene‐responsive element binding factor‐associated amphiphilic repression (EAR) motifs. Constitutive overexpression of *B1* in awned wheat produced an awnletted phenotype with pleiotropic effects on plant height and fertility. Transcriptome analysis of *B1* overexpression plants suggests a role as transcriptional repressor, putatively targeting pathways involved in cell proliferation. Haplotype analysis revealed a conserved *B1* coding region with proximal polymorphisms and supported the contention that *B1* is mainly responsible for awnletted wheats globally.
- *B1*, predominantly responsible for awn inhibition in wheat, encodes a C2H2 zinc finger protein with EAR motifs which putatively functions as a transcriptional repressor.

## Introduction

Awns are bristle‐like structures extending from the lemma‐tip‐midvein in the Poaceae grasses including cereal crop species such as wheat (*Triticum aestivum* and *T. durum*), barley (*Hordeum vulgare*) and rice (*Oryza sativa*). As an extension of the lemma, awns have long been considered modified leaves; however, evidence suggests that the origin or homology of the lemma itself may be as a modified sepal or novel organ type with bract and sepal characteristics (Grundbacher, 1963; Fabien & Hitoshi, 2015; Schrager‐Lavelle *et al*., 2017). In wild species, the main function of awns is grain dispersal by way of hygroscopically propelling the seed dispersal unit on the ground and into the soil (Elbaum *et al*., 2007). Furthermore, awns protect the grain from predation by animals or birds, especially if the awns are barbed. The barbs also aid in dispersal through attachment to animal fur (Hua *et al*., 2015). The grain dispersal functionality of awns has been lost during domestication in cereals due to inclusion of the nonshattering trait which is controlled by, amongst others, the *Tenacious glumes* (*Tg*) locus and the *Q* allele in wheat or *naked caryopsis* (*nud*) and *thresh‐1* genes in barley (Haas *et al*., 2019). Moreover, a general reduction in grain‐dispersal‐aiding appendages such as awns, barbs and hairs has accompanied domestication (Fuller & Allaby, 2009). In rice, breeding has artificially selected against awn presence or length because awns hinder harvesting and storage practices, and do not seem to contribute significantly to grain yield – likely related to the fact that rice awns lack chlorenchyma required for photosynthesis (Tatsumi & Kawano, 1972; Luo *et al*., 2013). In contrast to rice, wheat and barley awns have been retained to a great extent in domesticated cultivars, however the awns are shorter, thinner and lighter than those of their wild progenitors (Peleg *et al*., 2010; Haas *et al*., 2019). Awns in wheat and barley contribute to photosynthesis and can promote yield in warmer and drier rainfed environments ‐ as such awned wheats are prevalent in such areas as Australia, South and Central America, and the USA (Rebetzke *et al*., 2016). However, the absence of awns, often defined as awnletted due to minimal awn extension, also has been selected for particularly in wetter, more humid environments such as those in northern and central Europe where awns would not necessarily provide an adaptive advantage and may be a resource drain (Börner *et al*., 2005; Rebetzke *et al*., 2016).

Genes involved in awn development and elongation have been identified in rice and include: the basic helix‐loop‐helix transcription factor *Awn‐1* (*An‐1*) whose expression at the apex of lemma primordia causes continuous cell division for long awn formation (Luo *et al*., 2013); *Awn‐2* (*An‐2*) or *LONG AND BARBED AWN1* (*LABA1*) encoding Lonely Guy Like 6 (OsLOGL6), which catalyzes the final step of cytokinin synthesis and promotes awn elongation and growth (Gu *et al*., 2015; Hua *et al*., 2015); the YABBY transcription factor *DROOPING LEAF* (*DL*), which promotes awn formation in a noncell‐autonomous manner; the auxin response factor *OsETTIN* whose expression is required for awn development (Toriba & Hirano, 2014); and *REGULATOR OF AWN ELONGATION 2* (*RAE2*) or *GRAIN NUMBER, GRAIN LENGTH AND AWN DEVELOPMENT1* (*GAD1*), belonging to the EPIDERMAL PATTERNING FACTOR‐LIKE family of secretory peptides, whose peptide is cleaved by SUBTILISIN‐LIKE PROTEASE 1 to induce awn elongation (Bessho‐Uehara *et al*., 2016; Jin *et al*., 2016). Genes suppressing awn formation also have been identified: the YABBY transcription factor *TONGARI BOUSHI1* (*TOB1*) (Tanaka *et al*., 2012); and the mitogen‐activated protein kinase (MAPK) phosphatase, *GRAIN LENGTH AND AWN 1* (*GLA1*) (T. Wang *et al*., 2018), which negatively regulates both grain size and awn length. Other rice genes promoting awn development also are associated with grain number or length. For example, wild‐type (WT) *An‐1* or *RAE2/GAD1* alleles produce long awns, longer grains and reduce grains per panicle, whereas WT *An‐2/LABA1* alleles reduce grains per panicle. Similarly, in wheat and barley, awn presence or increases in awn length reduce grain number while increasing grain size (Schaller & Qualset, 1975; Rebetzke *et al*., 2016).

In barley, genes involved in brassinosteroid (BR) biosynthesis or signalling have been shown to affect awn length in addition to the multiple traits affected by BR including plant height, culm robustness and spike compactness (Dockter *et al*., 2014). The *Lks2* gene, underlying the *short awn 2* (*lks2*, for *length 2*) and allelic mutants *unbranched style 4* and *breviaristatum* (*ari‐d*), encodes a *SHORT INTERNODES* family transcription factor regulating awn length and pistil morphology (Yuo *et al*., 2012). Similar to wheat, where awn absence seems suited to moist environments, Yuo *et al*. (2012) suggest that the short‐awned *lks2* allele provides an adaptive advantage in the high‐precipitation areas of eastern Asia where natural variants of the allele originated. An intronic duplication in the homeobox gene *HvKnox3* produces the *Hooded* phenotype in which ectopic meristems develop on the lemma to form inflorescence‐like structures instead of normal awns (Müller *et al*., 1995; Williams‐Carrier *et al*., 1997).

In wheat, three dominant loci inhibiting awn development, *Tipped 1* (*B1*), *Tipped 2* (*B2*) and *Hooded* (*Hd*), have been identified and localized to chromosome arms 5AL, 6BL and 4BS, respectively (Watkins & Ellerton, 1940; Kato *et al*., 1998; Sourdille *et al*., 2002; Yoshioka *et al*., 2017). Wheat with dominant *B1* and *B2* genotypes produce small outgrowths of awns from the tip of the lemma, hence the terminology Tipped, and often are referred to as awnletted. However, in *B1* genotypes awns at the top of the head often reach 1 cm in length; while in *B2* genotypes awn length is more consistent with awns showing a slight curvature. Wheat with dominant *Hd* genotypes produces awns of reduced length that are pronouncedly curved with an inflexion from the base; there can also be membranous lateral expansions near the tip of the lemma which can resemble the *Hooded* phenotype from barley (Watkins & Ellerton, 1940; Yoshioka *et al*., 2017). *B1* is the most prevalent allele inhibiting awn development in both hexaploid and tetraploid wheats (Goncharov *et al*., 2003; Le Couviour *et al*., 2011; Mackay *et al*., 2014; Yoshioka *et al*., 2017). Despite the excellent progress in characterizing and mapping *B1*,* B2* and *Hd,* the genes responsible for awn inhibition at these loci have not been identified. Here, we report the identification of the *B1* awn inhibition gene through fine‐mapping using an F_2_ biparental durum wheat population, as well as a series of bread wheat mutants to delimit the genic region and identify the gene responsible for the awnletted trait. Furthermore, we characterize the identified *B1* gene, *TraesCS5A02G542800*, annotated as a C2H2 zinc finger gene, through constitutive gene overexpression in both durum and bread wheat to suggest a role as transcriptional repressor. Finally, haplotype analyses revealed *B1* diversity in wheat and were consistent with the assertion that *B1* is the most prevalent gene for awn inhibition. Our identification of *B1* parallels a companion paper in this issue by DeWitt *et al*. (2020), but whereas the present paper provides evidence for the repressor functionality of *B1,* DeWitt *et al*. (2020) detail the intricate relationship of *B1* and awn inhibition to grain length and spikelets per spike.

## Materials and Methods

### Plant materials and growth conditions

#### Durum wheat

Wheat plants were grown in 10‐ or 15‐cm pots containing Sunshine 8 (Sun Gro^®^ Horticulture, Agawam, MA, USA) mixed with a slow‐release fertilizer 14‐14‐14 in a sun room or glasshouse with approx. 16 h : 8 h, light : dark photoperiod and day/night temperatures of 22°C/18°C. To generate a durum wheat population segregating for awn presence, reciprocal crosses between the Canadian cultivar Strongfield (ST) and Australian Glossy Huguenot (GH, or AUS2499) were performed (Johnson *et al*., 1983; Clarke *et al*., 2005). F_1_ plants were self‐pollinated to produce an F_2_ population that consisted of 1936 plants, with 714 individuals grown in a glasshouse in 2016, 1135 grown in a glasshouse in 2017, and 87 F_2_ lines grown in a sunroom in 2016. F_1_ were also back‐crossed to ST to generate a BC_1_ population of 131 individuals in 2016. For awn phenotyping, F_2_ individuals were classified as fully awned or awnletted. Fully awned spikes had awn lengths > 1 cm throughout the entire spike whereas awnletted consisted of < 1 cm awns in the mid and basal spike regions; in some cases the apical part of an awnletted spike, especially the tip, had awn lengths of up to *c*. 3 cm. Additional phenotyping of the F_2_ population included measures of thousand kernel weight and grain size including area, length and width, performed using a Marvin seed analyzer (GTA Sensorik GmbH, Germany).

#### Hexaploid wheat

Spring wheat varieties Paragon and Cadenza are both awnless and carry the *B1* awn suppressor on chromosome 5A. Awned mutants were selected from three populations: a population of 6500 mutants of Paragon derived by ethyl methane sulfonate (EMS) treatment to induce nucleotide substitutions and developed to the M_6_ generation; a population of 2000 mutants in the Paragon background derived by gamma irradiation to produce deletions in DNA and developed to M_4_, (both populations were generated under the Defra‐funded Wheat Genetic Improvement Network at the John Innes Centre, Norwich, UK); and a population of 1200 mutants of Cadenza derived following EMS treatment, which has been re‐sequenced by exome capture and contains an estimated 9.0 million mutations. In addition, 258 wheat accessions or varieties (Supporting Information Table S6a) were screened using the primer sets designed for the awn‐suppressor candidate as described in the Supporting Information. Selected lines showing awn characteristics inconsistent with the PCR results were further characterized by sequencing and/or gene expression.

### Additional materials and methods

Details on the bulked segregant analysis, marker development and fine mapping, mutant deletion mapping, and transcriptome and haplotype analyses, with associated references can be found in Methods S1–S9 and Notes S1. The RNA‐seq data have been submitted to the Sequence Read Archive (SRA) at the NCBI with the accession numbers 11588058–11588082 (total RNA from *Triticum durum* and *Triticum aestivum*).

## Results

### Fine‐mapping the *B1* awn inhibitor in durum wheat

**Figure 1:**
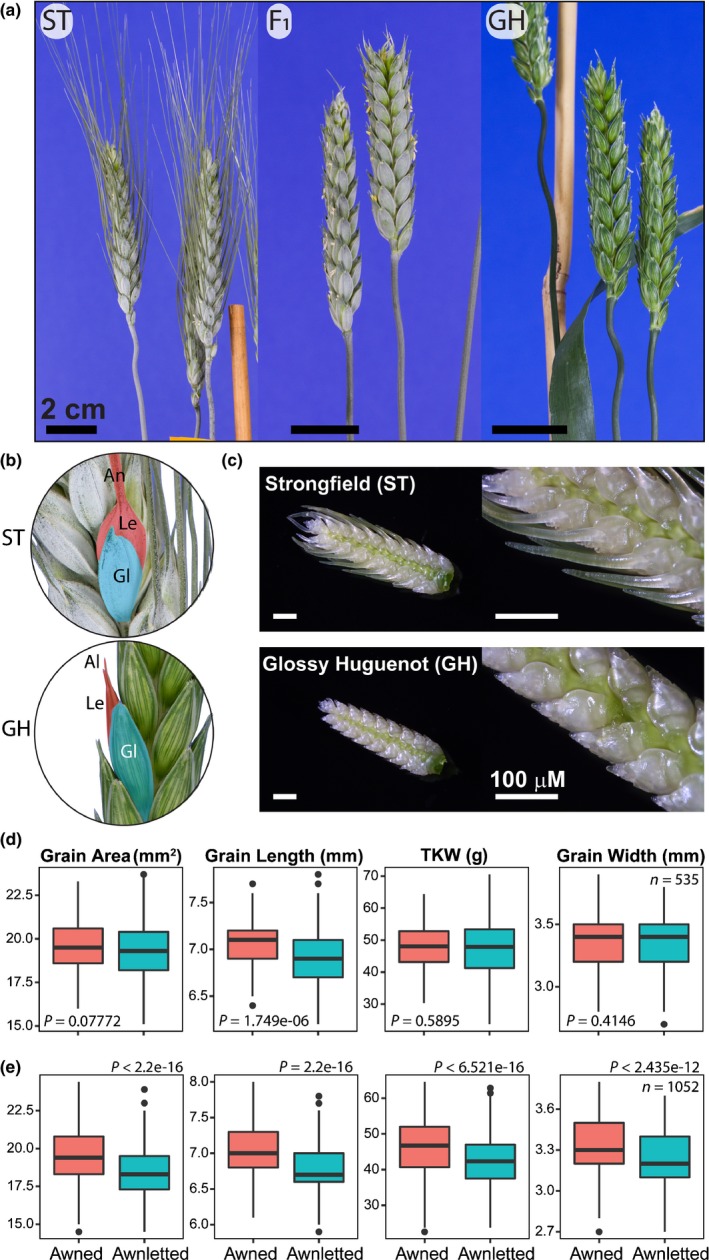
A durum wheat population segregating for awn presence. (a) Reciprocal crosses of the awned Strongfield (ST) and awnletted Glossy Huguenot (GH) cultivars were performed. All F_1_ plants were awnletted suggesting dominance over the awned trait. (b) The tip of the GH lemma (Le) was capped with an awnlet (Al), whereas the top of the glumes (Gl) were smooth and beakless. A predominant awn (An) extended from the tip of the lemma in ST whereas the top of the glume displayed a slight beak. (c) Awn growth was inhibited during early inflorescence development from the terminal spikelet stage during initial awn elongation. (d) In an initial glasshouse experiment in 15‐cm pots, awnletted lines of the STxGH F_2_ population were significantly reduced in grain length (*P* < 0.05). (e) In a second glasshouse experiment in 10‐cm pots, all measured grain traits including area, length, width and thousand kernel weight (TKW) were significantly reduced (*P* < 0.05) in awnletted lines of the STxGH F_2_ population. Boxplots of the grain trait data in (d, e) show the median (horizontal line), the interquartile range (boxes), 1.5‐times the interquartile range or maximum/minimum values (whiskers), and extreme values (dots). Red boxplots represent awned lines and blue boxplots represent awnletted lines of the GxH F_2_ population.

As a strategy to identify traits and genes associated with productivity under water stress, a biparental durum wheat population was developed from the cross of the Canadian cultivar Strongfield and the Australian Glossy Huguenot cultivar, which is further denoted as STxGH (Johnson *et al*., 1983; Clarke *et al*., 2005). The two cultivars differ in numerous traits including plant height, glaucousness, awn presence and grain size (Figs 1, S1). As demonstrated by the companion paper in this issue, DeWitt *et al*. (2020), and consistent with the previous studies in rice, barley and wheat discussed in the Introduction above, awn presence in STxGH promoted increases in grain size and in particular grain length (Fig. 1d,e). In terms of awn presence, Strongfield is fully awned whereas Glossy Huguenot is awnletted. In the F_1_ generation of the STxGH cross, the apical awns, around the top of the head, were longer in length suggesting that the awnletted trait is not completely dominant (Fig. 1a). However, for ease of phenotyping, phenotypes were grouped as fully awned or awnletted which included fully awnletted or awnletted awns of slightly longer length around the apical tip. With this phenotyping, plants segregated into awnletted : fully awned at a ratio of near 3 : 1 in the F_2_ population and 1 : 1 in the BC_1_ population, indicating that awn length is controlled by a single dominant inhibitor.

**Figure 2:**
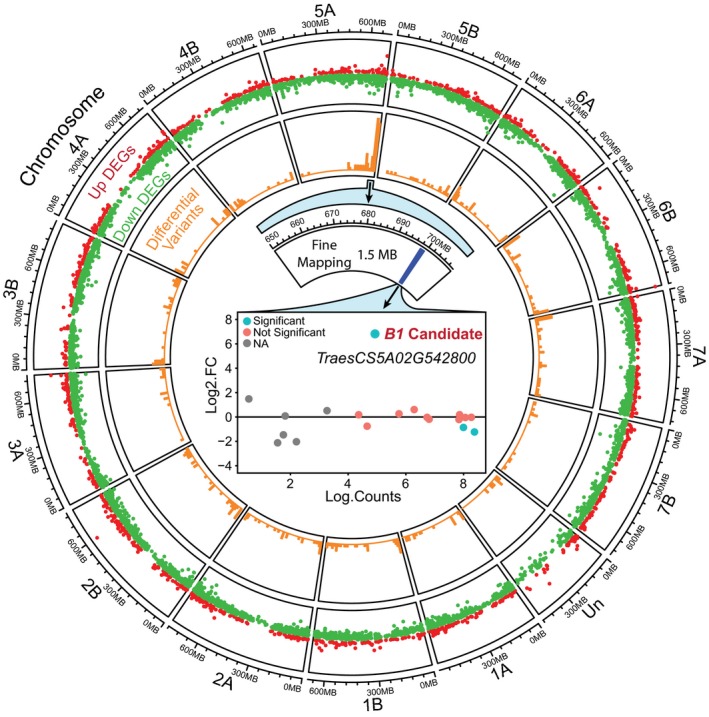
Identification of a candidate gene for the *Tipped1* (*B1*) awn inhibitor in durum wheat using bulked segregant RNA‐sequencing (BSR‐seq) and fine mapping. Circular tracks display chromosome name and size (MB, megabases) followed by differentially expressed genes (DEGs) and differential variants from BSR‐seq. Analysis of awned and awnletted BSR‐seq pools identified 6890 DEGs (*P*‐adjusted < 0.05) with the 5560 of these being downregulated in the awnletted pool. Peak differential variants were located on the distal end of chromosome 5A between 680 and 705 million‐nt in the Chinese Spring RefSeq 1.0 genome. Fine‐mapping using KASP markers delineated the genomic region responsible for the awn inhibitor trait to a *c*. 1.5‐Mbp region beginning at 698 003 657‐nt. Within this 1.5‐Mbp region, there were three DEGs: *TraesCS5A02G542600*,* TraesCS5A02G542800* and *TraesCS5A02G543000*. However, only *TraesCS5A02G542800* was upregulated in the awnletted pool and thus became the chief candidate for the *B1* awn inhibitor gene. Further, *TraesCS5A02G542800* was only expressed in the awnletted pool which is consistent with the model of a dominant inhibitor.

In order to fine‐map the awnletted trait bulked segregant analysis coupled with RNA sequencing (BSR‐seq) was performed using 714 F_2_ generation plants from the STxGH cross (Liu *et al*., 2012). The BSR‐seq pools, which consisted of *c*. 50–60 awned or awnletted lemmas or florets, were collected during swelling of the boot (Zadok stages 43–45). Following RNA sequencing and variant calling of the BSR‐seq pools, the greatest number of differential variants between the awned and awnletted pools were located on chromosome 5A and in particular the distal end of 5AL with a physical location between 680 and 705 million nt in the RefSeq 1.0 Chinese Spring genome reference (Figs 2, S2a,b; Table S1a) (International Wheat Genome Sequencing Consortium (IWGSC) *et al*., 2018). The dominant nature of the awnletted trait and 5A peak of differential variants between the awned‐awnletted pools is consistent with the location of the *B1* inhibitor locus (Yoshioka *et al*., 2017).

Following the discovery that the distal end of chromosome 5AL is associated with awn growth inhibition, 11 KASP markers were designed spanning the 670–708 million nt region on 5A for further fine‐mapping. A 652 F_2_ subset of the STxGH population was genotyped with the 11 KASP markers, and the markers were separated and ordered based on linkage analysis to create a local genetic map (Meng *et al*., 2015). Quantitative trait loci (QTL) mapping was performed with three different algorithms: inclusive composite interval mapping (ICIM), logistic regression for binary traits (LRB) and linear mixed model (LMM) (Meng *et al*., 2015; Broman *et al*., 2019). Significant QTL peaks for the awn trait were detected with logarithm of the odds (LOD) scores ranging from 377 to 128 (Fig. S2c). The QTL peaks were between markers *5A_B1_4* and *5A_B1_2* for ICIM and centred on marker *5A_B1* for LRB and LMM mapping with a Bayes credible interval of 48.73–58.43 cM, which spans the region from markers *5A_B1_4* to *5A_B1_2* (Fig. S2c). Marker *5A_B1* is based on the same polymorphism [A/C] as the published *B1* marker BobWhite_c8266_227 with different flanking sequences (Mackay *et al*., 2014). The single nucleotide polymorphism (SNP) markers *5A_B1_4* and *5A_B1_2* are physically located at 698 003 657 and 699 477 743 nt on chromosome 5A, which delimited the region containing the candidate gene for awn inhibition to *c*. 1.5 Mbp (Fig. 2).

In order to identify the gene responsible for awn inhibition on chromosome 5AL, the BSR‐seq data was analyzed for differentially expressed genes (DEGs, *P*‐adjusted < 0.05) between the awnletted and awned pools. There were 6890 DEGs (1330 up‐ and 5560 downregulated) between the two groups; however, there were only three DEGs, *TraesCS5A02G542600*,* TraesCS5A02G542800* and *TraesCS5A02G543000*, in the 1.5‐Mbp region between markers *5A_B1_4* and *5A_B1_2* (Fig. 2; Table S1b,c; Notes S2). Of the three DEGs, only *TraesCS5A02G542800* was upregulated in the awnletted pool and, moreover, showed no expression in the awned pool. The specific expression of *TraesCS5A02G542800* in the awnletted pool is consistent with the model of a dominant inhibitor. Thus, *TraesCS5A02G542800*, annotated as a C2H2 zinc finger encoding gene, became the chief candidate gene for awn inhibition in Glossy Huguenot and a suggested candidate gene for the *B1* awn inhibitor based on proximity to published markers for *B1* including *BobWhite_c8266_227* and *RAC875_C8642_231* (Mackay *et al*., 2014).

### Mapping mutations of the *B1* awn inhibitor in bread wheat

**Figure 3:**
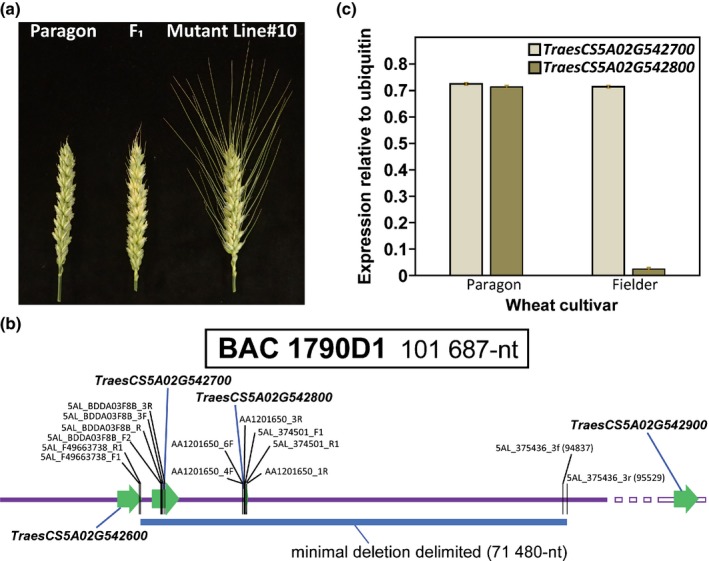
Utilizing deletion mutants in bread wheat to identify a candidate gene for the *Tipped1* (*B1*) awn inhibitor. (a) Dominant awn inhibition in the F_1_ generation, following a backcross of Paragon mutant line#10 to wild‐type Paragon. (b) Analysis of BAC 1790D1 from the French awned cultivar Renan confirmed mutant deletions for awn inhibition were confined to two genes, *TraesCS5A02G542700* and *TraesCS5A02G542800*. (c) Expression analyses of *TraesCS5A02G542700* and *TraesCS5A02G542800* revealed that only the expression of *TraesCS5A02G542800* was limited to the awnletted cultivar Paragon and thus *TraesCS5A02G542800* became the chief candidate for the *B1* awn inhibitor gene.

As part of the work to assess disease resistance between awned and awnletted wheat, mapping of mutations that caused an awned phenotype was used to identify the *B1* candidate gene. Three awned mutants within a Cadenza‐EMS TILLING population (1378awn, 1636awn and 1978awn) were identified (Krasileva *et al*., 2017). Three fully awned lines (1533, 1695 and 1987) also were identified in a Paragon‐EMS population. Because a full wheat reference genome was not available at the time, primers for genes in the awn inhibitor region on chromosome 5A were designed based on available wheat sequences and synteny to rice, *Brachypodium*, and sorghum (IWGSC1 + popseq genome assembly in Ensembl Plants, http://mar2016-plants.ensembl.org/Triticum_aestivum/Info/Index). During primer design, wheat A, B and D homoeologs were compared to produce A genome‐specific PCR markers. When the gene‐based chromosome 5A markers were used to screen the six awned mutants, it became clear that all six lines had lost significant portions of chromosome 5A in the region of the *B1* locus (Table 1). Awned Paragon line 1695 contained a smaller deletion than the other EMS‐treated awned lines. This delimited the awn‐suppressor to the region terminal to *TraesCS5A02G542600* (annotated as a hexose carrier encoding gene). The presence of large deletions in both EMS populations led us to screen a Paragon gamma‐irradiated population to seek lines carrying overlapping deletions to further define the region responsible for *B1* awn inhibition. In the Paragon‐Gamma population, the proportion of awned mutants was greater than in the EMS treated populations (eight awned lines among 2000 lines) as expected due to the nature of gamma irradiation, which tends to induce deletions > 10 kb (Morita *et al*., 2009). One awned mutant was identified from Paragon, line #10, which contained a small deletion spanning only two genes (Table 1). This Paragon awned mutant was backcrossed twice to the Paragon wild‐type (WT) to ensure that any additional mutations/deletions were removed and the mutation was consistent with the putative region for awn inhibition on chromosome 5AL. Similar to the durum crosses above in the STxGH population, it was evident that the awn inhibitor was dominant as the F_1_ plants all had short awns and plants segregated 3 : 1 for awnletted : fully awned in the F_2_ generation (Fig. 3a).

**Table 1:** Mapping of deletions in bread wheat.

IWGSC RefSeq v1.1	Position		Original gene name	Paragon gamma			Paragon EMS			Cadenza EMS		
	Start	Stop		#10	#11	#1	1695	1533	1987	1378awn	1636 awn	1978 awn
*TraesCS5A02G528400*	688662668	688666371	*Traes_5AL_CC97471EB*	nd	nd	nd						
*TraesCS5A02G529200*	688782883	688785747	*Traes_5AL_1A8232916*	nd	nd	nd		nd	nd		nd	nd
*TraesCS5A02G529700*	688944988	688953005	*Traes_5AL_3524CED56*	nd	nd	nd						
*no gene annotated*	689721892	689722407	*Traes_5AL_33F0FB676*	nd	nd	nd						
*TraesCS5A02G529900*	688973484	688976168	*Traes_5AL_74EEA8C04*	nd	nd	nd		nd	nd		nd	nd
*TraesCS5A02G531200*	689604419	689608548	*Traes_5AL_727C588D0*									
*TraesCS5A02G531800*	689896911	689912314	*Traes_5AL_D613D4F93*									
*no gene annotated*	690356469	690356927	*Traes_5AL_F212D729D*									
*TraesCS5A02G534100*	691436456	691441801	*Traes_5AL_9D148E259*					nd	nd		nd	nd
*TraesCS5A02G537000*	693625968	693632648	*Traes_5AL_69D2D5116*					nd	nd		nd	nd
*TraesCS5A02G540400*	697880912	697887556	*Traes_5AL_9F207C3A8*					nd	nd		nd	nd
*TraesCS5A02G541000*	698010055	698012286	*Traes_5AL_34CE42B51*									
*TraesCS5A02G542200*	698448290	698451049	*Traes_5AL_CD47A37A6*					nd	nd		nd	nd
*TraesCS5A02G542600*	698507247	698511217	*Traes_5AL_F49663738*					nd	nd		nd	nd
*TraesCS5A02G542700*	698512064	698517752	*Traes_5AL_BDDA03F8B*					nd	nd		nd	nd
***TraesCS5A02G542800***	**698528636**	**698529001**	***TRIAE_CS42_5AL_TGACv1_374501_AA1201650***					nd	nd		nd	nd
*non coding sequence*	698581401	698582186	*non coding sequence*					nd	nd	nd	nd	nd
*TraesCS5A02G542900*	698629158	698637157		nd	nd	nd	nd	nd	nd	nd	nd	nd
*no gene annotated*	698637620	698638706	*Traes_5AL_7F8E591BF*					nd	nd		nd	nd
*TraesCS5A02G543100*	698888618	698889913	*TRIAE_CS42_5AL_TGACv1_373973_AA1186000*					nd	nd		nd	nd
*TraesCS5A02G544900*	700347633	700351845	*Traes_5AL_55669B33*					nd	nd		nd	nd
*TraesCS5A02G546600*	700663097	700665985	*Traes_5AL_118352014*					nd	nd		nd	nd
*TraesCS5A02G547800*	702132455	702136920	*Traes_5AL_14CC809DB*									
*TraesCS5A02G550800*	704344835	704346995	*Traes_5AL_AB1566CF9*					nd	nd		nd	nd
*TraesCS5A02G551300*	704832604	704835391	*Traes_5AL_14402DE4B*									
*TraesCS5A02G552700*	705439246	705447278	*Traes_5AL_CFA8EDF16*	nd	nd	nd						
*TraesCS5A02G553300*	705842223	705847913	*Traes_5AL_92DEAA86D*	nd	nd	nd						
*TraesCS5A02G554100*	706235563	706237619	*Traes_5AL_1BE4452BD*	nd	nd	nd		nd	nd		nd	nd
*TraesCS5A02G554600*	706427023	706429950	*Traes_5AL_BBEF740F0*	nd	nd	nd						
*no gene annotated*	706436133	706438842	*Traes_5AL_BF079AF06*	nd	nd	nd						

Results of homoeologue specific PCR of genes on chromosome 5A. Red fill colour, gene amplicon absent; green fill colour, gene amplicon present; nd, not determined. The *B1* gene, *TraesCS5A02G542800*, is denoted in bold type.IWGSC, International Wheat Genome Sequencing Consortium.

A PCR designed from one of the potential candidate genes, *TraesCS5A02G542800*, was used to identify a bacterial artificial chromosome (BAC) clone (BAC 1790D1) from the French awned cultivar Renan that corresponded to the awn inhibitor sequence interval. The BAC sequence included three gene models *TraesCS5A02G542600*,* TraesCS5A02G542700* and *TraesCS5A02G542800*. It was shown that the sequence extended beyond the deletion of the Paragon awned mutant line #10 using a PCR primer set (Noncoding Scaffold 37546 3f/3r) which locates near the end of the BAC (Fig. 3b). Mapping of the deletions and utilization of the BAC sequence revealed two potential candidate genes, *TraesCS5A02G542700* and *TraesCS5A02G542800*, for awn inhibition. Next, RNA was isolated from developing spikes at the pre‐anthesis stage of the awned and awnletted varieties, Fielder and Paragon, respectively, and the expression of the two candidate genes was measured. The expression of *TraesCS5A02G542800* was limited to the awnletted cultivar whereas *TraesCS5A02G542700* was expressed to a similar level in both varieties (Fig. 3c). Thus, *TraesCS5A02G542800* was the chief candidate for *B1* in these UK bread wheats. Taken together with the BSR‐seq fine‐mapping of *B1* in durum wheat, it is proposed that *TraesCS5A02G542800*, annotated as a C2H2 zinc finger encoding gene, is the *B1* awn inhibitor gene – a statement also consistent with the companion paper by DeWitt *et al*. (2020).

### The B1 awn inhibitor TraesCS5A02G542800*,* a zinc finger protein with EAR motifs, belongs to a multigene family

**Figure 4:**
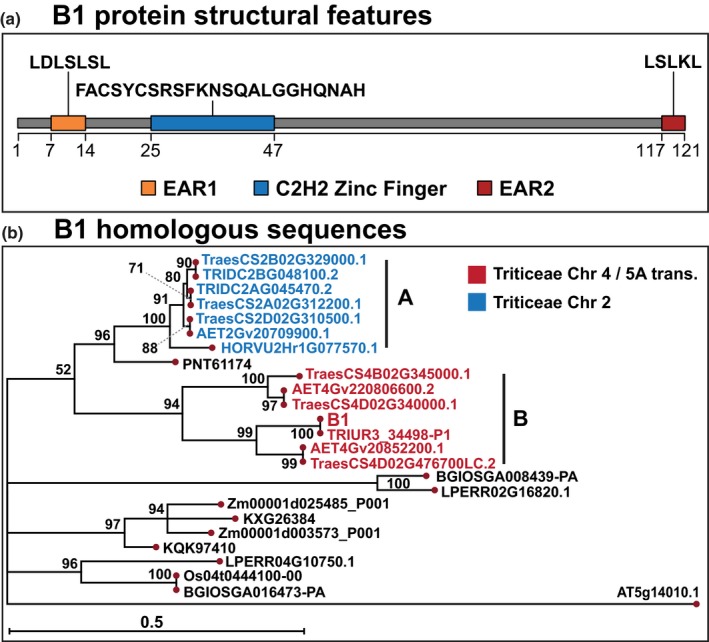
Tipped1 (B1) is a C2H2 zinc finger protein with ethylene‐responsive element binding factor‐associated amphiphilic repression (EAR) motifs and belongs to a multigene family. (a) Predicted structural features of the B1 protein including the location of a C2H2 zinc finger domain and two EAR motif sequences. (b) Maximum‐likelihood phylogeny of proteins with homology to B1. Triticeae species divide into two major grades: (A) B1‐related proteins from group‐2 chromosomes and (B) B1‐related proteins from group‐4 chromosomes. However, B1 has diverged from homoelogous proteins on chromosome 4B and 4D in sequence identity and may have undergone neo‐functionalization, perhaps due to the chromosome 4/5A translocation that occurred in the diploid progenitor of the A‐genome. TRIUR3_34498‐P1 from *Triticum urartu* was identical in sequence to B1; in wheat, the closest B1 homoelog was TraesCS4D02G476700LC with sequence identity of 74%. Protein sequences were downloaded from Ensembl Plants with identifiers retaining the isoform notation; full‐length proteins of TraesCS4D02G476700LC, TRIDC2AG045470, TRIDC2BG048100 and AET4Gv20806600 were identified as described (Supporting Information Fig. S3). Bootstrap support values from 1000 tests are shown; nodes were collapsed for bootstrap values < 50%; scale bar represents branch lengths as an estimation of the number of substitutions per amino acid. Species represented include: *Aegilops tauschii* (AET); *Triticum aestivum* (Traes); *T. dicoccoides* (TRIDC); *T. urartu* (TRIUR); *Hordeum vulgare* (HORVU); *Sorghum bicolor* (KXG); *Zea mays* (Zm); *Setaria italica* (KQK); *Oryza sativa* indica (BGIOSGA); *O. sativa* japonica (Os); *Leersia perrieri* (LPERR); and *Arabidopsis* *thaliana* (AT) (Methods S3).

The open reading frame (ORF) for the intron‐less *B1* awn inhibitor gene resides from 698 528 636 to 698 529 001‐nt on chromosome 5A in the RefSeq 1.0 Chinese Spring genome reference. The 122‐aa B1 protein contains a C2H2‐type zinc finger domain (residues 25–47) and two leucine‐rich ethylene‐responsive element binding factor‐associated amphiphilic repression (EAR) motif‐like sequences in its N‐terminal (LDLSLSL; residues 7–14) and C‐terminal (LSLKL; residues 117–121) regions (Fig. 4a) (Ohta *et al*., 2001; Kagale *et al*., 2010). The C2H2 family of zinc finger proteins is a large class of transcriptional regulators involved in a number of processes including trichome and root hair formation, flower development, seed development, disease resistance and abiotic stress response (Luo *et al*., 1999; Kazan, 2006; Xiao *et al*., 2009; Yan *et al*., 2014; K. Wang *et al*., 2018). Furthermore, the EAR motif is the most prevalent form of transcriptional repression motif identified in plants (Kagale & Rozwadowski, 2011). EAR‐domain‐containing proteins have been demonstrated to interact physically with corepressors, TOPLESS and SIN3 ASSOCIATED POLYPEPTIDE 18 (SAP18), and recruit chromatin remodelling factors to form a transcriptional repression complex.

In order to further characterize the B1 protein, Blast searches were performed of the Ensembl Plants database and ortholog, paralog, and homoeolog sequences in Ensembl were utilized to identify B1‐related amino acid sequences (Kersey *et al*., 2018). In addition, full‐length ORFs were identified from their respective genomes for the following genes: *TraesCS4D02G476700LC*,* TRIDC2AG045470*,* TRIDC2BG048100* and *AET4Gv20806600* (Avni *et al*., 2017; Luo *et al*., 2017; IWGSC *et al*., 2018). Following clustalw alignment, a maximum‐likelihood phylogeny was constructed to summarize relationships between B1 and sequences from Triticeae species *Aegilops tauschii,* barley, *Triticum dicoccoides* and *T. uratu* and additional members of the Class Liliopsida such as *Brachypodium distachyon, Leersia perrieri, Zea mays*,* Setaria italica, O. sativa* ssp. Indica and Japonica, and *Sorghum bicolor* (Figs 4b, S3) (Felsenstein, 1981; Jones *et al*., 1992; Thompson *et al*., 1994). Triticeae species divide into two major grades: (A) B1‐related proteins from group‐2 chromosomes and (B) B1‐related proteins from group‐4 chromosomes. The B1 awn inhibitor resides in the grade with group‐4 because the translocation T(4AL;5AL)1, a segmental interchange between chromosome arms 4AL and 5AL, occurred in the diploid progenitor of the wheat A subgenome (Dvorak *et al*., 2018; Grewal *et al*., 2018). The B1 protein shares important structural features with its homoeologs occurring on chromosome 4B (TraesCS4B02G345000) and chromosome 4D (TraesCS4D02G340000, TraesCS4D02G476700LC) but has diverged in sequence identity (58%, 60% and 74%, respectively) and promoter region, potentially leading to neo‐functionalization. Consistent with the companion paper by DeWitt *et al*. (2020), TraesCS4D02G476700LC shared the highest homology to B1 in wheat. However, TRIUR3_34498‐P1 from *T. urartu* was identical in sequence to B1. A paralog to B1, with 49% sequence identity, TraesCS2A02G312200, in the group‐2 chromosome grade, is orthologous to barley HORVU2Hr1G077570. *HORVU2Hr1G077570* is upregulated during overexpression of *FLOWERING LOCUS T3* (*HvFT3*); overexpression of *HvFT3* accelerated the initiation of spikelet primordia and resulted in upregulation of auxin related‐genes (Mulki *et al*., 2018). The GenBank entry ADK55064 for rice Os04g0444100 denotes that the C2H2 zinc finger MALFORMED SPIKELET is involved in specifying rudimentary glume identity (Zhang *et al*., 2011). The presence of EAR motifs in TraesCS5A02G542800 advocates for a function of transcriptional repressor and evidence is provided below through constitutive overexpression of *B1* that supports a repressor role.

### *B1* overexpression inhibits awn elongation with pleiotropic effects on plant development

**Figure 5:**
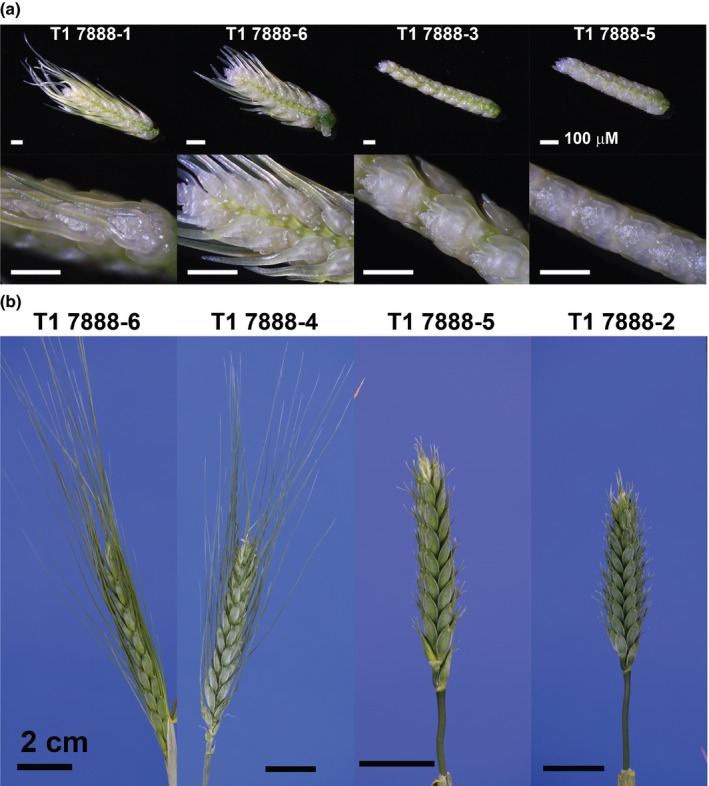
*Tipped1* (*B1*) overexpression in durum wheat represses awn formation throughout inflorescence development. (a) Awn development is inhibited during early awn extension when floral tissues are forming in transgenic lines expressing *B1* driven by the maize ubiquitin 1 promoter. In the T_1_ generation of line 7888, overexpression of *B1* segregates: awn development is not inhibited in lines with low expression of *B1* similar to wild‐type (WT) Strongfield and lines with high expression of *B1* display awn inhibition (Supporting Information Table S3). Bars, 100 μm. (b) Once flower heads fully emerge from the boot, T_1_ lines expressing *B1* displayed significant inhibition of awn growth with awnlets typically < 1 cm in length. Segregating T_1_ lines with low WT levels of *B1* expression were fully awned. Additional awn phenotypes of *B1* overexpression lines are shown in Figs S4–S7 for the durum wheat Strongfield and bread wheat Bobwhite backgrounds. Bars, 2 cm.

Introduction of the 366‐nt ORF of *B1* driven by the maize ubiquitin1 promoter into the durum cultivar Strongfield and bread wheat cultivar Bobwhite resulted in awn inhibition phenotypes (Figs 5, S4–S6; Table S2a–c). There were also additional pleiotropic phenotypes including reductions in plant height and floret fertility (Fig. S7; Table S2a–c). In some cases, T_0_ plants were totally infertile with no grain produced (Table S2a). Awn inhibition was heritable and was carried over into the T_1_ generation in both the Bobwhite and Strongfield backgrounds (Figs 5, S4).

### *B1* overexpression causes repression of auxin and cell proliferation genes

Young inflorescences collected following the terminal spikelet stage during early awn extension in Strongfield and Bobwhite WT plants (awned) were compared to awn inhibition in *B1* over‐expressing plants (awnletted) by performing RNA‐sequencing (Fig. 5a). Although overexpression through the ubiquitin promoter expanded the range of *B1* expression, as observed by the pleiotropic phenotypes, it was hypothesized that comparisons of the transcriptomes of *B1* over‐expressing plants would begin to reveal the molecular function of the *B1* awn inhibitor gene, including the possible role of B1 in transcriptional repression. Three RNA‐sequencing experiments were compared: (1) awnletted vs awned segregants of T_1_ Strongfield line 7888 (ST‐T1), (2) T_0_ Strongfield to WT (ST‐T0), and (3) Bobwhite T_0_/T_1_ awnletted lines to awned T_1_ line 7933 and Bobwhite WT (BW‐OE). In terms of overexpression of *B1*, ST‐T1 displayed the greatest average difference with log_2_ fold‐change (FC) of 7.4 and the ST‐T0 and BW‐OE with FC of 6.8 and 5.5, respectively (Table S3).

Gene ontology (GO) analysis was performed on the up‐ and downregulated DEGs from each awnletted‐awned comparison, ST‐T1, ST‐T0 and BW‐OE (Table S4). Common GO terms, for molecular function (MF), biological process (BP) and cellular compartment (CC), between the three comparisons were then selected (Table S4a). Significant GO terms (Fisher Classic, *P* < 0.05) for the upregulated DEGs included oxioreductase activity (MF), RNA glycosylase activity (MF), negative regulation of translation (BP) and extracellular region (CC). For the downregulated DEGs significant GO terms included: DNA binding transcription factor (TF) activity (MF), regulation of gene expression (BP), response to auxin (BP), and nucleus (CC). The effect of *B1* overexpression on GO with enrichment of TF activity, regulation of gene expression, and nuclear localization in the downregulated DEGs is consistent with the annotated functionality of B1 as a transcriptional repressor.

**Figure 6:**
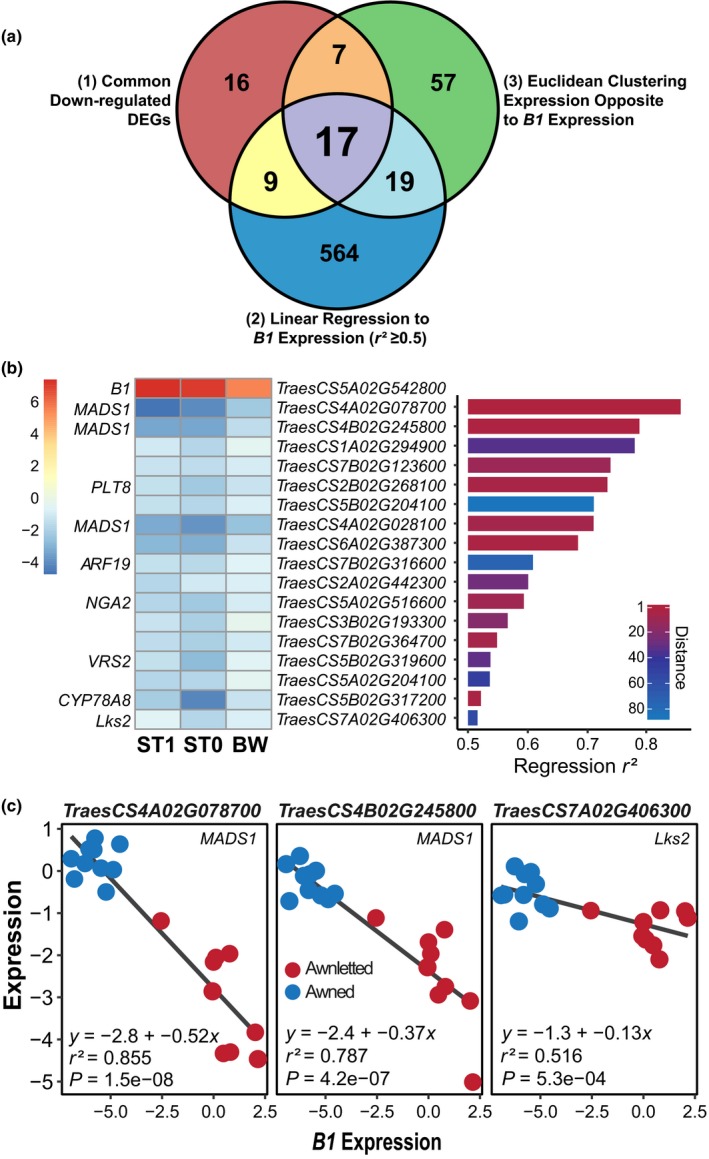
*Tipped1* (*B1*) overexpression in durum and bread wheat represses auxin‐ and cell‐proliferation‐related genes. (a) Venn diagram representing the relationships between: (1) common downregulated differentially expressed genes (DEGs) in the *B1* overexpression lines, (2) genes whose expression was linearly related to *B1* with *r* ^2^ ≥ 0.5, and (3) genes which were expressed in an opposite manner to *B1* as identified through Euclidean clustering of the additive inverse of log_2_‐normalized expression data (Supporting Information Fig. S8). (b) Seventeen genes were common to the three experimental analyses from (a). Within these, 17 were genes with orthology to *OsMADS1, OsPLT8, OsARF19, AtNGA2, HvVRS2, AtCYP78A8,* and *HvLks2* (*Os*,* Oryza sativa*;* At*,* Arabidopsis thaliana*;* Hv*,* Hordeum vulgare*). Heatmap represents the log_2_ fold‐change in expression between awnletted and awned inflorescences of Strongfield T_1_ and T_0_ and Bobwhite (ST1, ST0 and BW). Bar graph displays the *r* ^2^ value from the linear regression comparing the expression of the gene to *B1* expression; colours in the bar graph represent the Euclidean distance ranking from the clustering analysis. (c) Negative relationship of *TraesCS4A02G078700*,* TraesCS4B02G245800* and *TraesCS7A02G406300* expression to the expression of *B1* using linear regression (Table S5a). Expression is displayed as 75th percentile normalized. *P* represents *P*‐value.

In order to identify possible gene pathways that B1 might influence for awn inhibition and reduce possible artefacts from ubiquitous *B1* overexpression, a series of analyses was performed on the gene expression data (Fig. 6; Tables S3, S5). Because *B1* is annotated as a transcriptional repressor, which the GO analysis supported, the focus was on genes that were downregulated by *B1* overexpression. First, an analysis was made of the functional annotations of the DEGs common in at least two of the three experiments that included ST‐T1, ST‐T0 or BW‐OE (Table S3d). Second, to further narrow the list of putative genes or transcription factors that B1 may regulate, linear regression was performed comparing the expression of genes to the expression of *B1* in the over‐expression and control (WT) lines (Table S5a). Lastly, a clustering analysis was performed to identify genes expressed in an opposite pattern to *B1* (Table S5b; Fig. S8). From these analyses, 49 DEGs from the DESeq2 expression analyses were compared to 609 genes from linear regression analyses which had an *r* ^2^ value of ≥ 0.5 and the top 100 oppositely expressed genes compared to *B1* using the R/genefilter package (Fig. 6a; Table S5c) (Gentleman *et al*., 2018).

There were 17 genes common to the three analysis approaches used to identify potential pathways that could be regulated by B1 (Fig. 6a). These included genes associated with spike architecture, awn development and cell proliferation, and those participating in the auxin pathway (Figs 6b,c, S9). Within the gene set were MADS‐box genes *TraesCS4A02G078700, TraesCS4B02G245800* and *TraesCS4A02G028100* that have homology to rice *OsMADS1*, which is involved in meristem determinacy and differentiation and proliferation of the lemma and palea (Fig. S9a; Malcomber & Kellogg, 2004; Prasad *et al*., 2005). Transcription factors associated with auxin homeostasis included: (1) *TraesCS5B02G319600* orthologous to the barley *Six‐Rowed Spike* gene *VRS2*, a SHORT INTERNODES (SHI) transcription factor that contributes to auxin and cytokinin gradients during spike development (Fig. S9b; Youssef *et al*., 2017); (2) *TraesCS7B02G316600* which has homology to *AUXIN RESPONSE FACTOR 19* (*ARF19*), whose mutant in rice has been associated with developmental abnormalities of the palea and lemma (Fig. S9c; Zhang *et al*., 2016); and (3) *TraesCS2B02G268100,* with homology to *Arabidopsis thaliana* (Arabidopsis) *AINTEGUMENTA* (*ANT*) and rice *PLETHORA8* (*PLT8*). Members of the *AINTEGUMENTA*‐*LIKE*/*PLETHORA* (*AIL*/*PLT*) family in Arabidopsis are associated with auxin synthesis and signalling in both flower and root development (Krizek, 2011; Pinon *et al*., 2011; Mähönen *et al*., 2014). Also within the 17 genes common to the three analyses were: (1) *TraesCS5A02G516600*, a B3 domain transcription factor with homology to *NGATHA2* (*NGA2*) from Arabidopsis;* NGA* genes have been shown to be important regulators of auxin accumulation and distribution in developing Arabidopsis gynoeciums (Alvarez *et al*., 2009; Martínez‐Fernández *et al*., 2014) and (2) *TraesCS5B02G317200* with homology to the CYP78A class of cytochrome P450 genes such as *CYP78A8‐9* of Arabidopsis involved in promotion of cell proliferation during floral organ growth (Sotelo‐Silveira *et al*., 2013). However, perhaps the most relevant gene common to the three analyses of *B1* downregulated genes was *TraesCS7A02G406300*, the wheat gene orthologous to barley *Short Awn 2* (*Lks2*) (Fig. S9d). In barley, *Lks2* promotes awn elongation and has been proposed to affect auxin homeostasis (Yuo *et al*., 2012).

Overall, results of the gene expression analyses from the *B1* overexpression experiments suggest a role for the C2H2 zinc finger as transcriptional repressor. Furthermore, although overexpression through the ubiquitin promoter expanded the range of *B1* expression, and thus acted in tissues where it was not normally expressed, a common theme was the proportional downregulation of factors associated with cell proliferation and awn development.

### Polymorphisms adjacent to a conserved *B1* coding region are mostly predictive of *B1* awn inhibition

**Figure 7:**
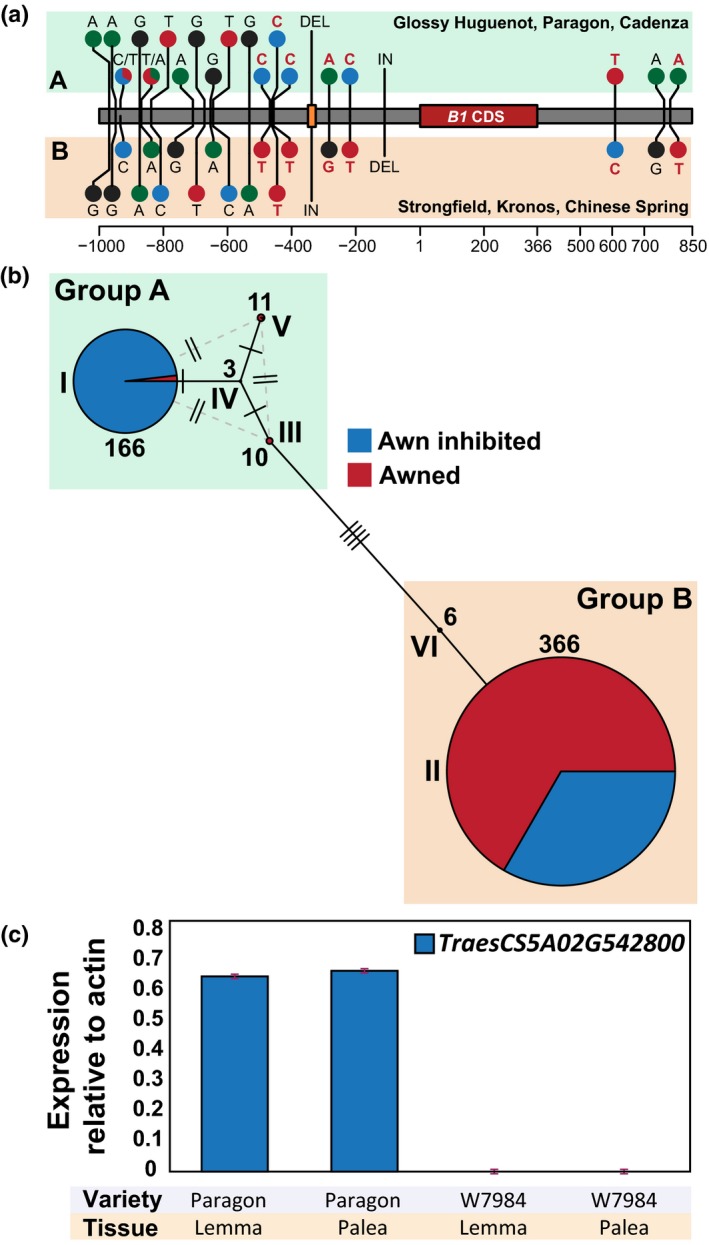
Haplotypes are mostly predictive of *Tipped1* (*B1*) awn inhibition. (a) Initial single nucleotide polymorphism (SNP) and InDel identification in the *B1* genomic region comparing awnletted‐*B1* (A) to awned‐*b1* (B) wheat accessions: Glossy Huguenot, Paragon and Cadenza to Strongfield, Kronos and Chinese Spring, respectively. There were no polymorphisms detected in the *B1* coding region. Seven markers used for haplotype screening are noted in red letters (Supporting Information Table S7). (b) Bread wheat accessions surveyed with KASP markers contain six different haplotypes which can be divided into two subgroups: Group A, in which 87% of accessions are awn‐inhibited, and Group B, of which 67% of accessions are awned, with the resulting 33% presumably containing a different awn inhibitor gene such as *Tipped2* (*B2*) or *Hooded* (*Hd*) (Table S6). In haplotype I, 98% of the accessions are awn‐inhibited indicating a functional *B1* inhibitor; however, associated haplotypes III, IV and V are mostly awned (96%). Pie graphs represent awn presence, Roman numerals represent haplotype group, numbers represent accessions in each haplotype group, and each perpendicular hash‐mark represents a SNP that was screened with a KASP marker. (c) Within Group A haplotypes, the synthetic hexaploid wheat W7984 is awned but does not express *B1* (Table S6).

In order to define possible polymorphisms important for *B1* function, haplotype analyses were performed across accessions of wheat from the UK, Canada and Asia. Genomic alignment of the *B1* region, 1000‐nt upstream and 850‐nt downstream of 698 528 636 to 698 529 001‐nt on chromosome 5A, identified SNPs, a 25‐nt deletion and a 1‐nt insertion that were present in genotypes with awn inhibition and revealed two major haplotype groups with a functional *B1* inhibitor (A) compared to a nonfunction *b1* (B) (Fig. 7a; Table S6). The polymorphisms were used to perform haplotype screens in diverse germplasm: (1) a PCR‐based screen to distinguish the 25‐nt polymorphism in a collection of 258 wheats including Watkins collection accessions (Wingen *et al*., 2014) and (2) KASP marker screening for select SNPs across a diverse collection of 562 wheats including Kyoto University accessions (Tanaka, 1983).

In the 258‐line collection, the PCR‐based screening of the 25‐nt deletion between awn and awnletted genotypes was highly predictive of awn inhibition (98%) (Table S6a). However, there were five exceptions that included the awned synthetic hexaploid wheat W7984 and awnletted Chinese Spring cultivar. In the 562‐line collection, KASP marker screening identified two major groups of haplotypes consistent with the sequence alignment data (Fig. 7b; Table S6b). Haplotype Group A was predominantly awn‐inhibited with a functional *B1* but contained a total of four haplotypes with the primary haplotype I containing 87% of the accessions. Ninety‐eight percent of haplotype I was awn‐inhibited, whereas minor haplotypes III, IV and V were 96% awned. Haplotype Group B was predominantly awned with a nonfunctional *b1* and contained one dominant haplotype II in which 67% of accessions were awned with the remaining 33% awn‐inhibited due to *B2*,* Hd*, or other inhibitory mechanisms (Fig. 7b; Table S6b). For example, it is known that Chinese Spring contains the *B2* and *Hd* awn inhibitors (Yoshioka *et al*., 2017). To confirm haplotypes identified from KASP marker screening in the 562‐line collection, a 1961‐nt *B1* interval from 29 accessions was sequenced, 1027‐nt upstream to 934‐nt downstream of the start codon of the *B1* gene (Table S6c). These *B1* interval sequences together with available genomic sequences revealed a conserved *B1* coding sequence and defined a total of nine haplotypes, six of which were within Haplotype Group A (Table S6c; Chapman *et al*., 2015; Avni *et al*., 2017; IWGSC *et al*., 2018; Clavijo *et al*., 2019). To further investigate the presence of awned accessions in Haplotype Group A, developing spikes from W7984 were isolated and it was found that *B1* was not expressed consistent with the awned phenotype observed in W7984 (Fig. 7c). The presence of awned accessions in haplotypes III, IV and V is intriguing and suggests that the mechanism responsible for *B1* expression is not present in these accessions. Sequencing analyses of the haplotype groups indicated that two polymorphisms are diagnostic for *B1* awn inhibition: an A/G polymorphism 709‐nt upstream from the *B1* start codon and an A/G polymorphism 761‐nt downstream of the start codon (Table S6c). However, the companion paper by DeWitt *et al*. (2020) suggests that no polymorphism within this region was diagnostic and that, rather, a 30‐nt deletion 4 kb downstream of the gene was the most (but not entirely) diagnostic for awn inhibition. Thus, the functional mechanism for *B1* expression and subsequent awn inhibition remains enigmatic and further experiments are required. For example, further analyses of the awned accessions in Group A, with predominantly *B1* haplotypes, are an important starting point for the discovery of the regulatory mechanisms for *B1* expression and awn inhibition.

## Discussion

Understanding the development of the inflorescence in cereal species is vital given the importance to yield and challenge for food security in the midst of climate change and population growth. In rice, numerous genes have been discovered and regulatory networks of inflorescence development have been defined (Zhang & Yuan, 2014; Chongloi *et al*., 2019). In barley and wheat, identification of regulators of inflorescence development is less mature but progressing given available genome sequences (Mascher *et al*., 2017; International Wheat Genome Sequencing Consortium (IWGSC) *et al*., 2018; Koppolu & Schnurbusch, 2019). Barley and wheat genes involved in inflorescence architecture have been discovered including: *VRS* genes involved in regulating two or six rows of grain in barley, wheat *TEOSINTE BRANCHED1* (*TB1*) involved in regulating inflorescence architecture and paired spikelet formation, or *Grain Number Increase 1* (*GNI1*) that contributes to wheat floret fertility (Komatsuda *et al*., 2007; Ramsay *et al*., 2011; Koppolu *et al*., 2013; Bull *et al*., 2017; Youssef *et al*., 2017; Dixon *et al*., 2018; Sakuma *et al*., 2019). The present study focused on the identification of the *Tipped1* (*B1*) awn inhibitor gene. Awns are an important component of wheat spike architecture, with their presence facilitating ancestral grain dispersal and influencing grain productivity through contributions to photosynthesis. Analyses of *B1*, encoding a C2H2 zinc finger protein, advocate for a function as transcriptional repressor and we provide evidence that suggests B1 may inhibit awn development by repressing pathways related to auxin and cell proliferation.

Auxin is a central regulator for primordium formation and cell proliferation (Czesnick & Lenhard, 2015; Huang & Irish, 2016; Wang & Jiao, 2017). During awn formation, a primordium forms at the tip of the lemma and this region has been suggested to be a ‘quasi‐meristem’ (Girin *et al*., 2009; Toriba & Hirano, 2014). In rice, formation of awn primordia is driven by auxin and characterized by enhanced cell division and proliferation (Luo *et al*., 2013; Toriba & Hirano, 2014). With relevance to *B1* function, although young inflorescences of constitutive overexpression plants were profiled and thus we cannot be certain of the precise genes B1 regulates during awn inhibition from the present results, there was an association of transcription factors related to auxin and cell proliferation. Furthermore, genes known to participate in awn and lemma development were among the genes downregulated in a proportional manner to *B1* overexpression. For example, the wheat ortholog of *length 2* (*Lks2*), a SHORT‐INTERNODES/STYLISH (SHI/STY) family transcription factor, was downregulated in unison with *B1* overexpression. Mutants of *Lks2* in barley are short‐awned and the *lks2* mutation has been suggested to reduce cell divisions in developing awns by affecting auxin concentrations (Yuo *et al*., 2012).

The genes that displayed the highest negative correlation to *B1* overexpression were orthologs of *Oryza sativa* (*Os*)*MADS1,* a key regulator of differentiation and proliferation of the lemma and palea in rice (Jeon *et al*., 2000; Prasad *et al*., 2005). The barley ortholog, *MADS‐box 7* (*HORVU4Hr1G067680*), is specifically expressed in the lemma and palea at the awn primordium stage (Schmitz *et al*., 2000). Interestingly, expression of an alternatively spliced *OsMADS1* is associated with increased grain length in rice (Liu *et al*., 2018). Liu *et al*. (2018) also demonstrated that OsMADS1 is a regulator of auxin synthesis, transport and response. Whether B1 regulates wheat *MADS1* genes for awn inhibition is unknown, but nonetheless the present data suggest a working hypothesis that *B1* expression inhibits genes involved in cell proliferation pathways required for awn development. As an analogy to this proposed B1 functionality, Arabidopsis KNUCKLES, a C2H2‐EAR‐motif protein with weak homology to B1, has been shown to repress the homeobox gene *WUSCHEL* to inhibit floral stem cell activity, helping to balance cell proliferation and differentiation in flower development (Sun *et al*., 2009).

Although it is suggested that *B1* acts as a transcriptional repressor, targeting genes related to cell proliferation, questions remain as to the specific genes that *B1* represses for awn inhibition. Analyses of transcriptomes of *B1* knockdown plants with varied expression levels will contribute to defining the true *B1* regulatory network. Furthermore, the tissues and cell layers, for example within the lemma, which express *B1* in awned and awnletted wheat need to be established. Experiments examining the distribution of auxin, through reporter assays such as *DR5::GUS*, should be performed in awned and awnletted wheat (Ulmasov *et al*., 1997). Questions remain, but we nonetheless present the identification of the *B1* awn inhibitor gene and its phylogeny and haplotype history. Furthermore, we provide evidence linking *B1* to suppression of awn formation through transcriptional repression laying the groundwork to further explore B1 functionality including interacting protein partners and possible downstream targets that cause awn inhibition.

## Author contributions

DH, AJC, CAM, PN and JAF designed the research; DH, QZ, TM, YB, DJFK, MM, MC, NMA, CC and AS performed the research; DH, FMY, SK, CAM, PN and JAF analyzed the data; and DH, SK, NMA, PN, CAM and JAF wrote the paper.

## Supporting information

Please note: Wiley Blackwell are not responsible for the content or functionality of any Supporting Information supplied by the authors. Any queries (other than missing material) should be directed to the *New Phytologist* Central Office.

**Fig. S1** Phenotypes of the parental cultivars and F_1_ offspring of Canadian cultivar Strongfield (ST) and the Australian cultivar Glossy Huguenot (GH).
**Fig. S2** Fine‐mapping of awn trait in STxGH F_2_ population.
**Fig. S3** clustalw alignment of B1 and related proteins.
**Fig. S4** *B1* overexpression in the bread wheat cultivar Bobwhite inhibits awn growth.
**Fig. S5** *B1* overexpression consistently represses awn growth in T_0_ plants.
**Fig. S6** Inflorescence phenotypes of *B1* overexpression lines at maturity.
**Fig. S7** Reduction in plant height and awn length resulting from overexpression of *B1* gene.
**Fig. S8** Workflow to identify the top 100 *B1* similarly and oppositely expressed genes in *B1* overexpression lines.
**Fig. S9** Amino acid alignments of wheat proteins with orthology to MADS1, VRS2 and Lks2, whose encoding genes were differentially regulated in *B1* overexpression lines.
**Fig. S10** Association of KASP markers *5A_AL7* and *5A_AL1* to awn inhibition.
**Methods S1** Bulked segregant analysis with RNA sequencing (BSR‐seq) in durum wheat.
**Methods S2** Gene overexpression in durum and bread wheat.
**Methods S3** Statistical analyses, data mining and data visualization.
**Methods S4** Marker screening and QTL mapping in durum wheat.
**Methods S5** Mapping *B1* in hexaploid awned mutants.
**Methods S6** BAC library screening.
**Methods S7** Gene expression studies by quantitative PCR.
**Methods S8** Characterizing wheat varieties for the *TraesCS5A02G542800* haplotypes.
**Methods S9** *B1* mapping in hexaploid wheat biparental RIL population of BW278 and AC Foremost.
**Notes S1** Association of two KASP markers to awn inhibition in durum and bread wheat.
**Notes S2** Differentially expressed genes from BSR‐seq demonstrate awns are a photosynthetic organ.

**Table S1** Bulked segregant RNA‐sequencing (BSR‐seq) analyses.

**Table S2** Phenotypes of *B1* overexpression plants.

**Table S3** Differentially expressed genes in *B1* overexpression plants.

**Table S4** Gene ontology analyses of differentially expressed genes in *B1* overexpression lines.

**Table S5** Identification of genes with expression profiles similar or opposite to *B1* expression.

**Table S6** Haplotype analysis of *B1* genomic region in diverse wheat germplasm.

**Table S7** Primers used in the present study.
