# Supplementary material for: Dominant inhibition of awn development by a putative zinc‐finger transcriptional repressor expressed at the B1 locus in wheat
Source: New Phytol. 2019 Oct 10;225(1):340–55. doi: 10.1111/nph.16154 (PMC6916588; doi:10.1111/nph.16154)
Supplement: Supplementary file 1 — Fig. S1 Phenotypes of the parental cultivars and F1 offspring of Canadian cultivar Strongfield (ST) and the Australian cultivar Glossy Huguenot (GH). Fig. S2 Fine‐mapping of awn trait in STxGH F2 population. Fig. S3 clustalw alignment of B1 and related proteins. Fig. S4 B1 overexpression in the bread wheat cultivar Bobwhite inhibits awn growth. Fig. S5 B1 overexpression consistently represses awn growth in T0 plants. Fig. S6 Inflorescence phenotypes of B1 overexpression lines at maturity. Fig. S7 Reduction in plant height and awn length resulting from overexpression of B1 gene. Fig. S8 Workflow to identify the top 100 B1 similarly and oppositely expressed genes in B1 overexpression lines. Fig. S9 Amino acid alignments of wheat proteins with orthology to MADS1, VRS2 and Lks2, whose encoding genes were differentially regulated in B1 overexpression lines. Fig. S10 Association of KASP markers 5A_AL7 and 5A_AL1 to awn inhibition. Methods S1 Bulked segregant analysis with RNA sequencing (BSR‐seq) in durum wheat. Methods S2 Gene overexpression in durum and bread wheat. Methods S3 Statistical analyses, data mining and data visualization. Methods S4 Marker screening and QTL mapping in durum wheat. Methods S5 Mapping B1 in hexaploid awned mutants. Methods S6 BAC library screening. Methods S7 Gene expression studies by quantitative PCR. Methods S8 Characterizing wheat varieties for the TraesCS5A02G542800 haplotypes. Methods S9 B1 mapping in hexaploid wheat biparental RIL population of BW278 and AC Foremost. Notes S1 Association of two KASP markers to awn inhibition in durum and bread wheat. Notes S2 Differentially expressed genes from BSR‐seq demonstrate awns are a photosynthetic organ. [file NPH-225-340-s001.pdf]

New Phytologist Supporting Information

Article title: **Dominant Inhibition of Awn Development by a Putative Zinc-Finger Transcriptional Repressor Expressed at the *B1* Locus in Wheat**

Authors: **Daiqing Huang, Qian Zheng, Tancey Melchkart, Yasmina Bekkaoui, David JF Konkin, Sateesh Kagale, Martial Martucci, Frank M You, Martha Clarke, Nikolai M Adamski, Catherine Chinoy, Andrew Steed, Curt A McCartney, Adrian J Cutler, Paul Nicholson and J Allan Feurtado**

Article Acceptance Date: 16 August 2019

The following supporting information is available for this article:

Methods S1: Bulked segregant analysis with RNA sequencing (BSR-seq) in durum wheat

Methods S2: Gene overexpression in durum and bread wheat

Methods S3: Statistical analyses, data mining and data visualization

Methods S4: Marker screening and QTL mapping in durum wheat

Methods S5: Mapping *B1* in hexaploid awned mutants

Methods S6: BAC library screening

Methods S7: Gene expression studies by quantitative PCR

Methods S8: Characterizing wheat varieties for the *TraesCS5A02G542800* haplotypes

Methods S9: *B1* mapping in hexaploid wheat bi-parental RIL population of BW278 and AC Foremost

Notes S1: Association of two KASP markers to awn inhibition in durum and bread wheat

Notes S2: Differentially expressed genes from BSR-seq demonstrate awns are a photosynthetic organ

Figure S1: Phenotypes of the parental cultivars and F_1 offspring of Canadian cultivar Strongfield (ST) and the Australian Glossy Huguenot cultivar (GH).

Figure S2: Fine-mapping of awn trait in STxGH F_2 population.

Figure S3: ClustalW alignment of B1 and related proteins.

Figure S4: *B1* overexpression in the bread wheat cultivar Bobwhite inhibits awn growth.

Figure S5: *B1* overexpression consistently represses awn growth in T_0 plants.

Figure S6: Inflorescence phenotypes of *B1* overexpression lines at maturity.

Figure S7: Reduction in plant height and awn length resulting from overexpression of *B1* gene.

Figure S8: Workflow to identify top one-hundred *B1* similarly and oppositely expressed genes in *B1* overexpression lines.

Figure S9: Amino acid alignments of wheat proteins with orthology to MADS1, VRS2, and Lks2, whose encoding genes were differentially regulated in *B1* overexpression lines.

Figure S10: Association of KASP markers *5A_AL7* and *5A_AL1* to awn inhibition.

SI References

Table S1. Bulked segregant RNA-sequencing (BSR-seq) analyses.

Table S2. Phenotypes of *B1* overexpression plants.

Table S3. Differentially expressed genes in *B1* overexpression plants.

Table S4. Gene ontology analyses of differentially expressed genes in *B1* overexpression lines.

Table S5. Identification of genes with expression profiles similar or opposite to *B1* expression.

Table S6. Haplotype analysis of *B1* genomic region in diverse wheat germplasm.

Table S7. Primers used in this study.

Methods S1: Bulked segregant analysis with RNA sequencing (BSR-seq) in durum wheat

Lemma or young floret tissues were collected from spikes following swelling of the boots (Zadoks 45) but prior to ear emergence (Zadoks 49) from F_2 plants and pooled into three pairs of awned or awnletted bulks in which we performed BSR-seq (Liu *et al.*, 2012). Each bulk consisted of samples from 50-60 individual F_2 plants. Total RNA was isolated using the mirVana miRNA kit with Plant RNA Isolation Aid (Thermo Fisher Scientific) according to the manufacturer's protocol. RNA was treated with DNase using the TURBO DNA-free™ Kit (Thermo Fisher Scientific) to remove traces of DNA. RNA quality was measured by the Agilent BioAnalyzer 2100 with RNA 6000 Nano kit (Agilent Technologies) according to the manufacturer's instructions. The TruSeq Stranded mRNA library synthesis kit (Illumina, Incorporated) was used according to manufacturer instructions to create sequencing libraries and sequencing was carried out on an Illumina HighSeq 2500 to obtain paired-end 125-nt reads. A variety of tools in the commercial software CLC Genomics Workbench 11 (Qiagen Bioinformatics) was used to process and analyze the RNA sequencing data. The RNA sequencing reads were trimmed to remove the sequencing adaptors and sequences with low quality. The cleaned RNA reads were mapped to the A and B sub-genomes of the International Wheat Genome Sequencing Consortium (IWGSC) reference sequence v1.0 assembly of Chinese Spring using CLC Genomics Workbench 11 (Qiagen Bioinformatics) with a setting of length fraction at 0.8 and similarity fraction at 0.8 (IWGSC *et al.*, 2018).

For analysis of variants in the BSR-seq pools, BAM files from three awned RNA-seq pools were merged and the BAM files from the three awnletted RNA-seq pools were merged. SNP calling was performed on the merged awned and awnletted BAM files using the basic variant detection tool in the CLC Genomics Workbench 11 to create variant tracks with the setting of minimum coverage of 10 and minimum count at 2 and minimum frequency of 5%. Differential variants between awned and awnletted pools were identified by filtering against the control reads using the variant comparison workflow in CLC Genomics Workbench 11 (awned as control to awnletted). The differential variants with control coverage < 10 were filtered out. For differential variant analysis, differential variants per chromosome were normalized to the chromosome length before plotting. To localize the differential variants on chromosome 5A, differential variants were plotted against the chromosomal location using histogram bins of 10 million-nt. For gene expression analyses, reads were trimmed with Trimmomatic 0.35 and pseudo-aligned using Kallisto 0.42.4 using the IWGSC RefSeq 1.1 transcripts from the A and B sub-genomes available at Ensembl Plants (Bolger *et al.*, 2014; Bray *et al.*, 2016; Kersey *et al.*, 2018). For differential gene significance testing, Kallisto data was imported into R using the tximport package and

significance testing was carried out using the DESeq2 package using the recommended workflows (Love *et al.,* 2014; Soneson *et al.,* 2015).

Methods S2: *B1* overexpression in durum and bread wheat

Cloning of the *B1* open reading frame (ORF) was accomplished using DNA from the Glossy Huguenot cultivar since the gene lacks introns. *B1* was PCR-amplified using the high-fidelity-enzyme PfuUltra II Fusion HS DNA polymerase (Agilent Technologies) and cloned into the Gateway cloning vector pDONR221 using the Gateway BP Clonase II (Thermo Fisher Scientific). The *B1* ORF in pDONR221 was transferred into the pANIC 5D vector (Mann *et al.*, 2012) using LR Clonase II (Thermo Fisher Scientific) following the manufacturer's protocol. The pANIC 5D vector contains the *ZmUbi1* promoter (maize ubiquitin 1 promoter and intron) to drive the expression of *B1* and a selectable marker, bar gene, which was placed under the transcriptional control of the rice actin 1 gene (*OsAct1*) promoter. The plasmid DNAs of pANIC 5D-B1 was transformed into the durum awned wheat Strongfield and awned bread wheat Bobwhite through Biolistic-mediated transformation of wheat as described in Huang *et al.* (2017).

For RNA-sequencing of *B1* overexpression lines, developing inflorescences were collected when awn primordium and floral tissues were forming as shown in Fig. 5a, Fig. S4b. RNA extraction, sequencing, processing of the reads, and gene expression analyses were performed as described above for BSR-seq including use of the Kallisto-DESeq2 workflow.

Methods S3: Statistical analyses, data mining and data visualization

The R statistical computing language (version 3.5.1), running within the RStudio integrated development environment (version 1.1.463), was used for significance testing, data analysis and production of graphs and figures; this included integration of the Bioconductor software framework for many of the tasks as outlined below (Huber *et al.,* 2015; R Core Team, 2018; RStudio Team, 2018). The following R packages also contributed to the analysis of data: dplyr (Wickham *et al.*, 2018a); readr (Wickham *et al.*, 2018b); tidyr (Wickham and Henry, 2018); plyr (Wickham, 2011). In particular, dplyr was used heavily within the R environment to arrange data and select common elements between dataframes. Graphs, except as noted, were produced using the package ggplot2 (Wickham, 2016). For some datasets, preliminary data arrangement and summary occurred in Microsoft Excel 2013. PDF figure files from R were arranged and edited in Adobe Illustrator CC (version 22.1). Adobe Photoshop CC (version 19.1.8) was used to arrange photographs of plants and inflorescences of *B1* overexpression lines.

To visualize differentially expressed genes and differential variants, a circular plot was constructed using the circlize R package (Gu *et al.,* 2014). An annotation R package, org.Taestivum.eg.db, was created by downloading wheat, rice, and *Arabidopsis* data from Ensembl Plants BioMart using the biomaRt package and database assembly with the AnnotationForge package (Durinck *et al.,* 2009; Kinsella *et al.,* 2011; Carlson and Pagès, 2018). The org.Taestivum.eg.db database was used to assign annotations to gene lists and rice and *Arabidopsis* annotations from BioMart were added with the dplyr R package. Gene ontology analysis was performed according to Klaus and Huber (2016) using the genefilter, geneplotter, and topGO R packages (Alexa and Rahnenfuhrer, 2018; Gentleman and Biocore, 2018; Gentleman *et al.,* 2018). Venn groups and heatmaps were constructed using the Venndiagram and pheatmap, respectively (Chen and Boutros, 2011; Kolde, 2018). To find similarly expressed genes to *B1*, counts normalized by library size were exported from DESeq2, the data from all overexpression experiments combined, and counts which equaled zero were changed to one count. Data was transformed to log2 and normalized by subtracting the median and dividing by the mean average deviation (median-MAD) of each sample (Fig. S8). The genefilter R package was used to find the top 100 similarly expressed genes, to *TraesCS5A02G542800*, based on Euclidean distance. To find genes oppositely expressed to *B1*, the same procedure as finding similarly expressed genes was implemented except that the additive inverse of the median-MAD normalized data was used before proceeding to the genefilter R package. For calculation of linear models of the top 100 oppositely expressed genes, log2-transformed and 75th-percentile-normalized expression data was using in calculation of the linear model for each gene compared to *B1* (Fig. 6c, Table S5a). The phylogenetic tree of proteins with homology to *B1* was constructed with CLC Genomics Workbench 11 (Qiagen) using the ClustalW alignment algorithm and maximum likelihood phylogeny with the neighbor-joining method and JTT protein substitution and 1000 bootstrap tests (Felsenstein, 1981; Jones *et al.*, 1992; Thompson *et al.*, 1994). Proteins for the phylogenetic tree were downloaded from Ensembl Plants for the following species: *Aegilops tauschii, Arabidopsis thaliana, Brachypodium distachyon, Hordeum vulgare*, *Leersia perrieri, Oryza sativa* subspecies Indica and Japonica, *Sorghum bicolor*, *Triticum dicoccoides*, *Triticum uratu,* and *Zea mays* (Arabidopsis Genome Initiative, 2000; Yu *et al.*, 2002; International Rice Genome Sequencing Project, 2005; Paterson *et al.*, 2009; International Brachypodium Initiative, 2010; Schnable *et al.*, 2012; Avni *et al.*, 2017; Ling *et al.*, 2017; Luo *et al.*, 2017; Mascher *et al.*, 2017; Stein *et al.*, 2018). Identification of full-length open reading frames occurred for truncated proteins utilizing the appropriate genome sequences in CLC Genomic Workbench 11 (Qiagen Bioinformatics) (Fig. S3). To visualize variants adjacent to the *B1* gene region, the lolliplot function of the trackViewer package was used (Ou *et al.,* 2019). The haplotype figure, Fig. 6b,

was constructed using the R package pegas with data from Table S6b (Paradis, 2010). Tests of statistical significance of grain size parameters were tested with the Wilcoxon rank sum test in R.

Methods S4: Marker screening and QTL mapping in durum wheat

Tissue for DNA extraction was collected from young seedling leaves (2.5 cm sections) for each F_2 plant. The samples were freeze-dried in a 96-well plate (Qiagen #19560) for two days in a Labconco Freeze Dry System FreeZone6 and then ground to a fine powder in a mini beadbeater-96 using three to four 2-mm Zirconia beads (BioSpec Products, Inc.) for 2 min. DNA was extracted using an automated DNA extraction protocol using the AGP965 Plant DNA Extraction Kit on an AutoGenprep 965 machine (AutoGen, Holliston, MA, USA) following the manufacturer's protocol. DNA concentration was measured using a Synergy H1 Hybrid Multi-Mode Reader with Take3 Plate (Biotek). DNA concentrations were normalized to 200 ng/μL for Kompetitive allele specific PCR (KASP) genotyping.

For marker design, selected variants (SNPs/MNP (multi-nucleotide polymorphism)/InDels) within the *B1* QTL region were converted into KASP markers (LGC group, Biosearch Technologies). The 100 bp flanking genomic sequences of each variant was extracted from wheat genomic sequence using CLC Genomic Workbench 11 (Qiagen Bioinformatics) and saved as a fasta file with the variant information. The SNP markers were converted to genome-specific KASP assays using PolyMarker, which circumvents the limitations associated with the polyploid wheat genome and generates allele-specific primers for a specified sub-genome (Ramirez-Gonzalez *et al.*, 2015). The allele-specific primers were designed carrying the standard FAM (5′ GAAGGTGACCAAGTTCATGCT 3′) and VIC (5′ GAAGGTCGGAGTCAACGGATT 3′) tails with the targeted SNP at the 3′ end. A common primer was designed with the total amplicon length < 120-nt.

SNP genotyping of the F_2 population with KASP markers was performed using high-throughput Fluidigm Integrated Fluidic Circuit (IFC) in the formats of 48.48 (2,304 reactions) or 192.24 (4,608 reactions) in a Fluidigm BioMark HD (Fluidigm Corporation) according to the Biomark HD Data Collection User Guide (PN 100-2451 K1). The assay mix and sample mix were prepared according to the protocol for SNP Genotyping on FR48.48 Dynamic Array IFCs and the protocol for SNP Genotyping on 192.24 Dynamic Array IFCs. All reagents and SNP genotyping related products such as IFCs for kompetitive allele-specific polymerase chain reaction (PCR) were ordered from Fluidigm (https://www.fluidigm.com/) except the 2x KASP Master Mix (KBS-1016-002) ordered from LGC group, Biosearch Technologies. Genotyping followed Wang *et al.* (2009) and data were analyzed using the Fluidigm SNP Genotyping Analysis Software.

To prepare the genotyping and phenotyping data for QTL mapping and perfect marker screening, the data was organized using the R packages plyr, dplyr and tidyr and plotted with ggplot2. For QTL mapping, genotypes were filtered for 'No Call' and only calls with confidence ≥ 80 percent were retained. For diagnostic marker screening, genotypes with 'Invalid' or 'No Call' were filtered out of the dataset for analysis and plotting. QTL mapping was performed using inclusive composite interval mapping (ICIM) in the BIP (bi-parental populations) model of QTL IciMapping software v4.0 (Meng *et al.*, 2015) with the P values for entering variables (PIN) = 0.05. The threshold of the logarithm of the odds (LOD) scores for evaluating the statistical significance of QTL effects was determined using 1000 permutations at the significance level of 0.05. QTL mapping was also performed using the r/qtl2 package using genome scans for binary traits by logistic regression and using a genome scan with linear mixed model (Broman *et al.*, 2019).

Methods S5: Mapping *B1* in hexaploid awned mutants

Preliminary mapping of the awn suppressor locus was undertaken using three wheat populations segregating for the presence of awns (data not shown). A previous study reported that the SNP marker *BobWhite_c8266_227* from the Illumina Infinium iSelect 90,000 SNP wheat array (http://www.illumina.com/) was strongly associated with the presence of awns and that the awn-suppressor interval could be defined as lying between SNP markers *wsnp_Ex_c20899_30011827* and *RAC875_c61559_435* in an interval of 7.5 cM (Mackay *et al.*, 2014). The DNA sequences associated with these markers were used to interrogate the wheat sequence in the IWGSC1+popseq database within Ensembl Plants to identify associated genes. In the absence of a sequenced wheat genome when initiating this study the awn suppressor interval was compared across *Brachypodium distachyon*, rice and sorghum using sequences from the Ensembl Plants database (http://plants.ensembl.org/index.html). Genes showing conservation of synteny across the interval in the three species were selected to develop markers from the wheat orthologs. The A, B and D genome homoeologs of hexaploid wheat were compared and PCR primers developed to amplify specifically from the A genome. Primer sets were used to screen DNA from parents and awned mutants of the three populations. As new releases of the wheat genome sequence became available additional PCR markers were developed to refine the awn-suppressor interval. A list of the PCR primers is provided in Table S7b.

Methods S6: BAC library screening

The Renan BAC library, Tae-B-Renan, held by CNRGV was screened for identifying *TraesCS5A02G542800*-bearing clones residing at the awn suppressor *B1* locus. The 2D pooling strategy used for organizing the

BAC library allowed screening in two steps. Firstly, PCR screening of the 2D pools allows the identification of the plates containing a specific clone of interest. Then, for each positive plate, secondary row and column pools are then produced and screened to identify a positive clone. (https://cnrgv.toulouse.inra.fr/Services/Screening-services/DNA-Pool-production). The primer pair 5AL_374501_F1 and AA1201650_1R was used for screening (Table S7a). Real Time PCR amplification was carried out in a total volume of 20 μL consisting of 5 μL of Sigma SYBR Green JumpStart Taq ReadyMix, 0.05 μL of both the forward and the reverse primer (50 μM), and 2 μL of 2D pool DNA. PCRs were performed on a CFX96 Touch™ Real-Time PCR Detection System (Bio-Rad) using the following cycling protocol: 95 °C for 10 min followed by 40 cycles of 95 °C for 20 s and 60 °C for 1 min. A dissociation curve with light measurement every 0.1°C was included to confirm amplification of single gene products.

Methods S7: Gene expression studies by quantitative PCR

Total RNA was prepared from tissue using Qiagen RNeasy Plant Mini Kit according to the manufacturer's protocol and subsequently treated with TURBO DNA-free™ Kit (Thermo Fisher Scientific) to remove residual genomic DNA contamination. First-strand cDNA was synthesized using Superscript III 1st strand RT-PCR system (Thermo Fisher Scientific) according to manufacturer's instructions. qRT-PCR was carried out in a total volume of 20 μL consisting of 10 μL of Sigma SYBR Green JumpStart Taq ReadyMix, 1 μL of both the forward and the reverse primer (10 μM), and 4 μL of 1:5 diluted template cDNA solution. Primers for *TraesCS5A02G542800* were 5AL_374501_F1 and 5AL_374501_R1. Primers for *TraesCS5A01G542700* were 5AL_BDDA03F8B_3F and 5AL_BDDA03F8B_3R (Table S7a). Relative quantitation of gene expression was carried out using the $2^{-\Delta\Delta CT}$ method (Livak and Schmittgen, 2001). The wheat ubiquitin and actin genes were used as endogenous references to normalize expression levels of target genes. Primers for ubiquitin were PB67_Ubiquitin_F and PB68_Ubiquitin_R and for actin were PB47_Actin_F and PB48_Actin_R (Table S7.1). Three independent amplifications were performed from each cDNA sample, and reactions were done in triplicates. PCR was performed on a CFX96 Touch™ Real-Time PCR Detection System (Bio-Rad) using the following cycling protocol: 95 °C for 10 min followed by 40 cycles of 95 °C for 15 s and 60 °C for 1 min. A dissociation curve was included to confirm amplification of single gene products.

Methods S8: Characterizing wheat varieties for the *TraesCS5A02G542800* haplotypes

For screening a collection of 258 wheat accessions, two PCR primer sets were designed to characterize wheat varieties on the basis of the 25-nt insertion-deletion by gel-based separation. One set

AA1201650_4F and AA1201650_3R (4f/3rev) differentiates on the basis of product size producing a product of 483 bp from wheat accessions lacking the 25-nt insertion and a product of 507 bp from wheat accessions carrying the 25-nt insertion. The second set AA1201650_6F and AA1201650_4R (6f/4rev) produces a product of 287 bp only from varieties carrying the 25-nt insertion. The PCR cycling conditions were: 95°C for 15 min followed by 35 cycles of 94°C for 30 sec, annealing at 61°C for 4f/3rev or 68°C for 6f/4rev for 30 sec, 72°C for 30 sec. A final step of 72°C for 10 min was included before samples were held at 10°C prior to gel analysis.

For a KASP marker-based haplotype analysis of 562 wheat accessions, 9 SNP variants adjacent to the *B1* gene were converted into KASP markers (*5A_AL1* to *5A_AL9*, Table S7a). The KASP genotyping assays were performed in a total volume of 5 μL, which contained DNA (37.5 ng/2.5 μL), KASP Assay Mix (0.07 μL), and 2.5 μL 2x KASP Master Mix (V4.0, LGC Genomics). Genotyping PCR reactions were conducted on a DNA Engine Dyad Thermal Cycler (Bio-Rad Laboratories, Incorporated). The cycling conditions were: Hot-start activation at 94°C for 15 min (1 cycle), and ten touchdown cycles (94°C for 20 s; touchdown 61°C, -0.6°C per cycle, 60 s) and then 26 cycles of 94°C for 20 s and 55°C for 60 s, 3 cycles of 94°C for 20 s and 57°C for 60 s, hold at 4°C. The reaction plates were then read for the fluorescent signals of FAM, HEX and ROX using an Omega Fluorostar plate reader (BMG LABTECH GmbH, Ortenberg, Germany). The KASP plate reader data were converted to allele calls using KlusterCaller software (LGC Genomics, Beverly USA). For sequencing based haplotype analysis (Table S6.3), primers ZF6F3 and ZF6R3 were used to amplify *B1* and flanking sequences and the amplicons were PCR purified for Sanger sequencing. The sequencing data were analyzed using CLC genomics software 11 (Qiagen). All primers are listed in Table S7.1. Additionally, for haplotype analysis, available genomic sequences were downloaded and the *B1* region on chromosome 5A was aligned with CLC genomics software 11 (Qiagen). Sequence data from the 10+ Genome Project (http://www.10wheatgenomes.com/) of the bread wheats Cadenza, Paragon, Robigus, and Claire, and the durum Kronos were made available pre-publication (Clavijo *et al.*, 2019) having been previously assembled using the w2rap method (Clavijo *et al.*, 2017a,b).

Methods S9: *B1* Mapping in hexaploid wheat bi-parental RIL population of BW278 and AC Foremost

Preliminary linkage analysis of the *B1* awn inhibitor in the Superb/BW278 doubled haploid population (n=142) located *B1* between the proximal SNP BS00023152_51 (690,548,798 bp on CS RefSeq v1.0; KASP *kwm1037*) and the distal SNP Tdurum_contig8348_831 (700,443,596 bp on CS RefSeq v1.0; KASP *kwm1036*). BW278 is a Canadian wheat breeding line that carries the *B1* awn inhibitor, while Superb is a Canadian wheat variety that is awned. Using the KASP assays *kwm1037* and *kwm1036*, 192 RILs were

selected from the BW278/AC Foremost RIL population (n=1,872) that had crossover events between the proximal SNP BS00023152_51 (*kwm1037*) and the distal SNP Tdurum_contig8348_831 (*kwm1036*). AC Foremost is a Canadian wheat variety that is awned. Awn phenotype was determined by growing these RILs to the heading stage in a greenhouse. A RIL was either fully awned or apically awnletted. Intermediate phenotypes were not observed in these RILs since they were homozygous at loci in the *B1* interval. KASP assays were designed for wheat Infinium 90K assay SNPs that were polymorphic on BW278 and AC Foremost, and had BLAST hits in the *B1* interval defined by *BS00023152_51* and *Tdurum_contig8348_831* (Table S7c). In addition, exome capture sequence data from BW278 and AC Foremost was also mined for SNPs. These KASP assays were tested on the 192 selected RILs from the BW278/AC Foremost RIL population. Linkage analysis was conducted with MapDisto v.1.7.7 (Lorieux 2012).

Notes S1: Association of two KASP markers to awn inhibition in durum and bread wheat

Two KASP markers, *5A_AL7* and *5A_AL1*, were used to genotype a second greenhouse growth experiment of the STxGH durum population. *5A_AL1* was 99.4% and *5A_AL7* was 99.9% diagnostic for differentiating the awn-awnletted trait (Fig. S10a). We further confirmed the co-segregation of *5A_AL1* and *5A_AL7* markers with *B1* awn inhibition in the Canadian spring wheat RIL population BW278 x AC Foremost (awnletted x awned) that consisted of 192 lines and had been previously used for fine-mapping of the *B1* awn inhibitor gene (Fig. S10b).

Notes S2: Differentially expressed genes from BSR-seq demonstrate awns are a photosynthetic organ

We looked for commonalities between the down-regulated DEGs from the BSR-seq experiment and the down-regulated DEGs from the *B1* overexpression (*B1*-OE) experiments. There were 7 DEGs in common and this low number is likely related to the significant differences in stages, inflorescences during swelling of the boot (Zadok stages 45-49) in BSR-seq versus early spike development when floral organ patterning was occurring in *B1*-OE. Furthermore, the BSR-seq did not reciprocate the differences in auxin-related genes as noted for the *B1*-OE experiments (Table S1b, S3a-c). Instead, GO analyses revealed significant terms associated with photosynthesis, an expected result given the role of awns in photosynthesis (Table S1c; Maydup *et al.*, 2010). Interestingly, cytokinin response was also significantly associated with awns and up-regulation of gibberellin 20-oxidase biosynthesis and GA-2-ox catabolism genes was observed, a predictable result in comparing awnletted lemmas with growth-arrested awns to lemmas with actively extending awns (Table S1b).

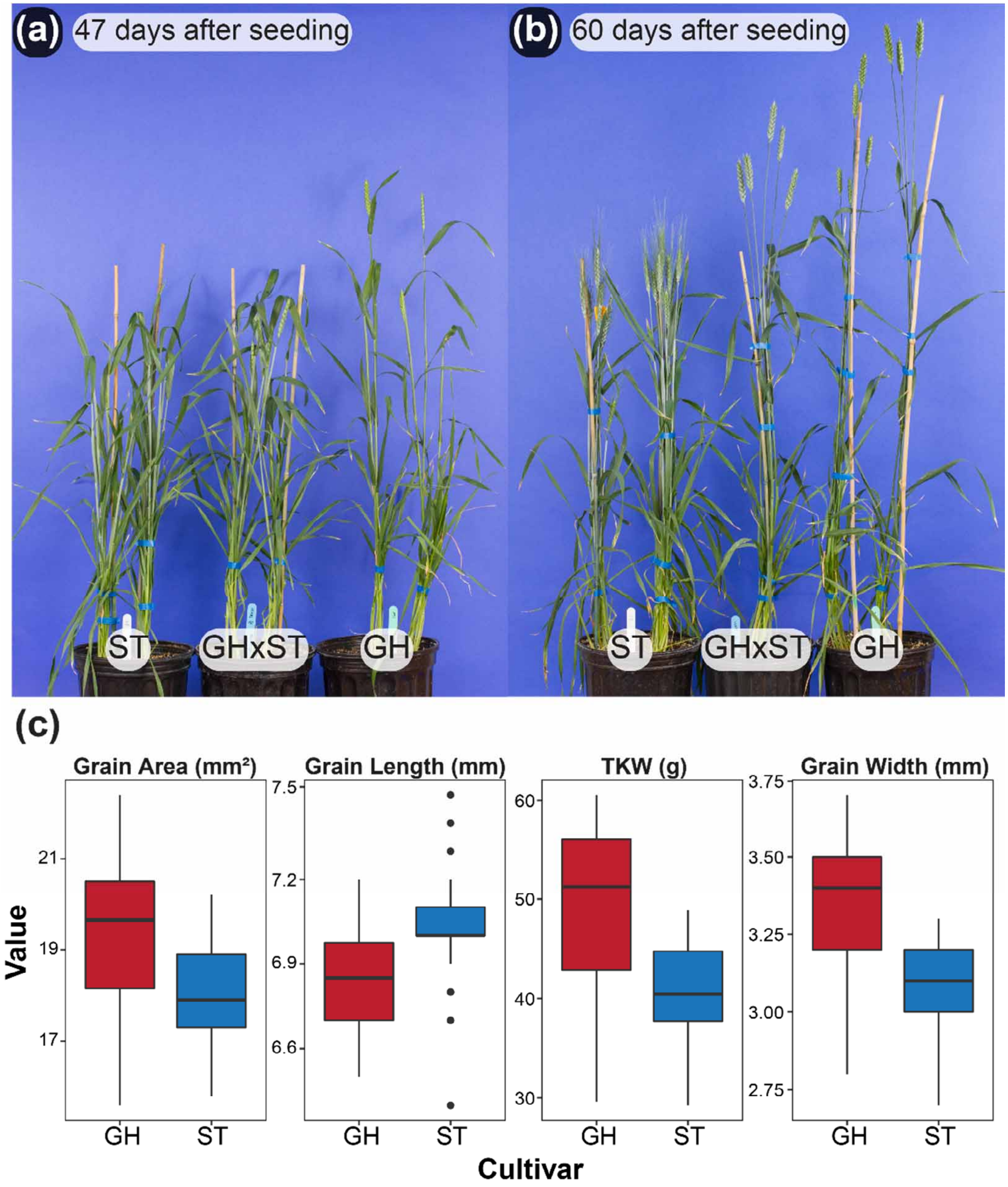

Figure S1. Phenotypes of the durum wheat parental cultivars and F_1 offspring of Canadian cultivar Strongfield (ST) and the Australian Glossy Huguenot cultivar (GH).
(a) Differences in plant height and heading time between parents and F_1 at 47 days after seeding. (b) Differences in plant height, awn presence, head type between the parents and F_1 at 60 days after seeding. (c) Grain size traits of parental cultivars GH and ST; 42 and 26 wheat heads were analyzed for the grain traits in GH and ST, respectively. Boxplots of the grain trait data show the median (horizontal line), the interquartile range (boxes), 1.5-times the interquartile range or maximum / minimum values (whiskers), and extreme values (dots). Red boxplots represent GH and blue boxplots represent ST. TKW represents Thousand Kernel Weight.

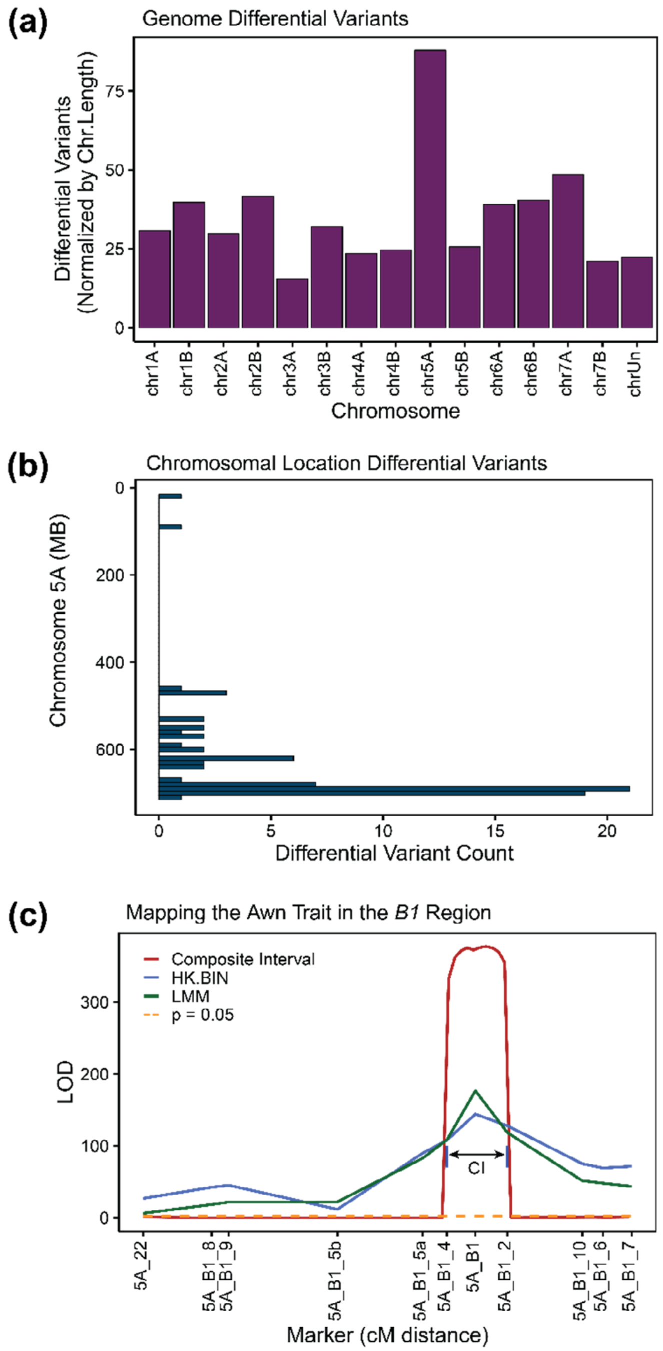

Figure S2. Fine-mapping of awn trait in STxGH F_2 durum wheat population.
(a) Distribution of differential variants (SNPs, MNPs and InDels) between F_2 awned and awnletted RNA pools indicating chromosome 5A contained the most differential variants. (b) Differential variants are enriched at the distal end of chromosome 5A between 680-705 million-nt in the RefSeq 1.0 Chinese Spring genome reference. (c). Using 11 KASP markers designed from SNPs in the 680-705 million-nt chromosome 5A genomic region, the awn trait was fine-mapped to a 1.5 MB (Megabase) genomic region between marker *5A_B1_4* and *5A_B1_2* and centered on marker *5A_B1* using three different algorithms: inclusive composite interval mapping (Composite Interval, red), logistic regression for binary traits (HK.BIN, blue) and linear mixed model (LMM, green). CI represents confidence interval.

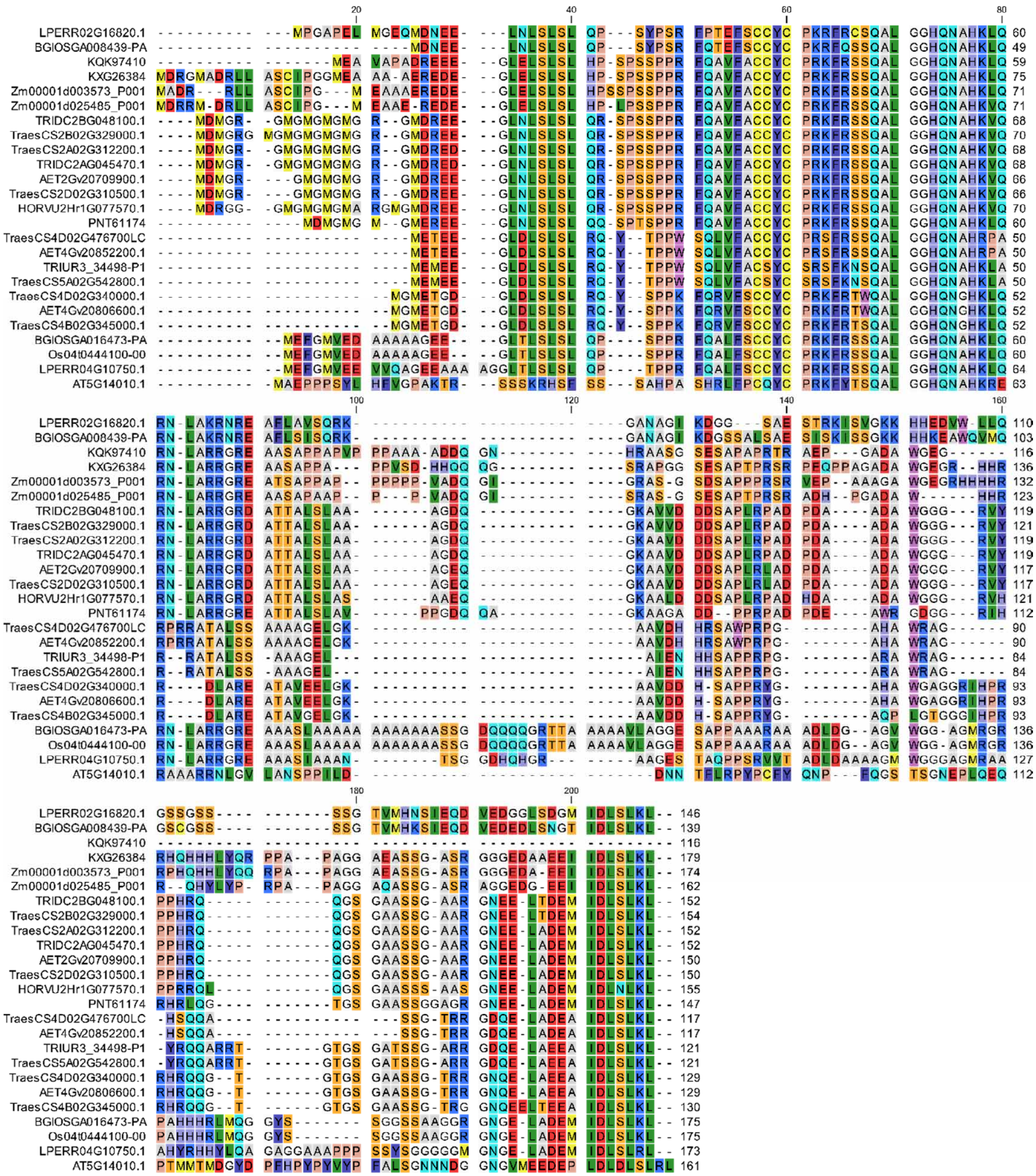

Figure S3. ClustalW alignment of B1 and related proteins.

Proteins with homology to B1 were identified through BLAST searches of the Ensembl Plants database and by utilizing ortholog, paralog, and homoeolog tools in Ensembl (Kersey et al., 2018). Full-length open reading frames were identified from their respective genomes for the following genes: *TraesCS4D02G476700LC*, *TRIDC2AG045470*, *TRIDC2BG048100*, and *AET4Gv20806600* (Avni *et al.*, 2017; Luo *et al.*, 2017; IWGSC *et al.*, 2018). Species represented include: *Aegilops tauschii* (AET); *Triticum aestivum* (Traes); *T. dicoccoides* (TRIDC); *T. urartu* (TRIUR); *Hordeum vulgare* (HORVU); *Sorghum bicolor* (KXG); *Zea mays* (Zm); *Setaria italica* (KQK); *Oryza sativa* indica (BGIOSGA); *O. sativa* japonica (Os); *Leersia perrieri* (LPERR); Arabidopsis (AT) (Methods S3).

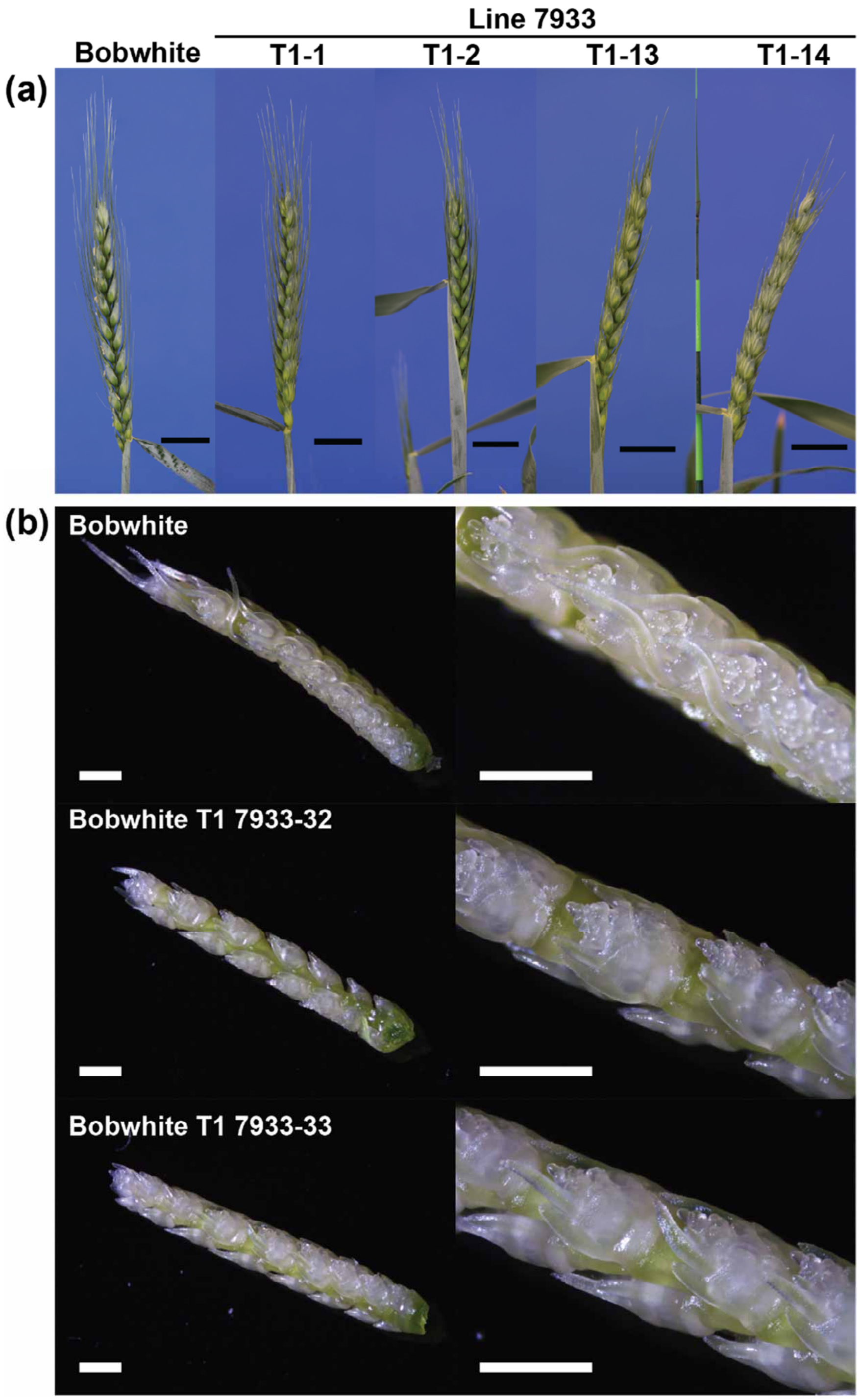

Figure S4. *B1* overexpression in the bread wheat cultivar bobwhite inhibits awn growth.
(a) During head emergence, awn length segregated in T_1 generation of *B1* overexpression line 7933. Lines T1-1 and T1-2 showed similar awn length to parent Bobwhite while T1-13 and T1-14 showed reduced awn length. Scale bar represents 100 μm. (b) Awn development was inhibited from early in development when awn primordium and floral tissues were forming in *B1* overexpression line 7933 T_1 plants. T_1 lines 7333-32 and 7333-33 showed inhibited awn development compared to Bobwhite wildtype. Scale bar represents 2 cm.

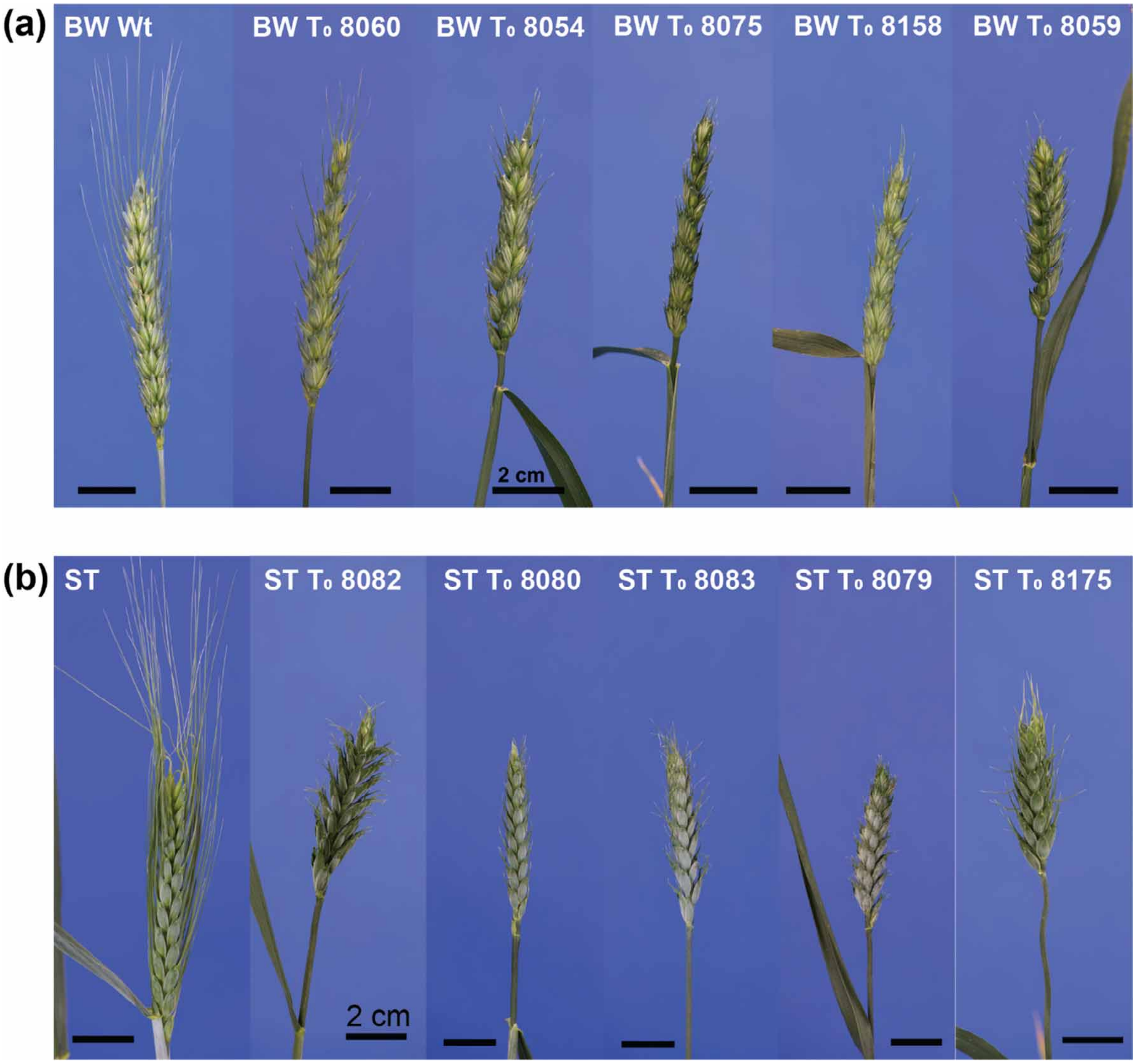

Figure S5. *B1* overexpression consistently represses awn growth in T_0 plants.
(a) Inhibition of awn growth in T_0 plants of the Bobwhite (BW) bread wheat cultivar compared to wildtype. (b) Inhibition of awn growth in T_0 plants of the Strongfield (ST) durum wheat cultivar compared to wildtype. Line names are denoted in the above figure following BW T_0 or ST T_0. Scale bar represents 2 cm.

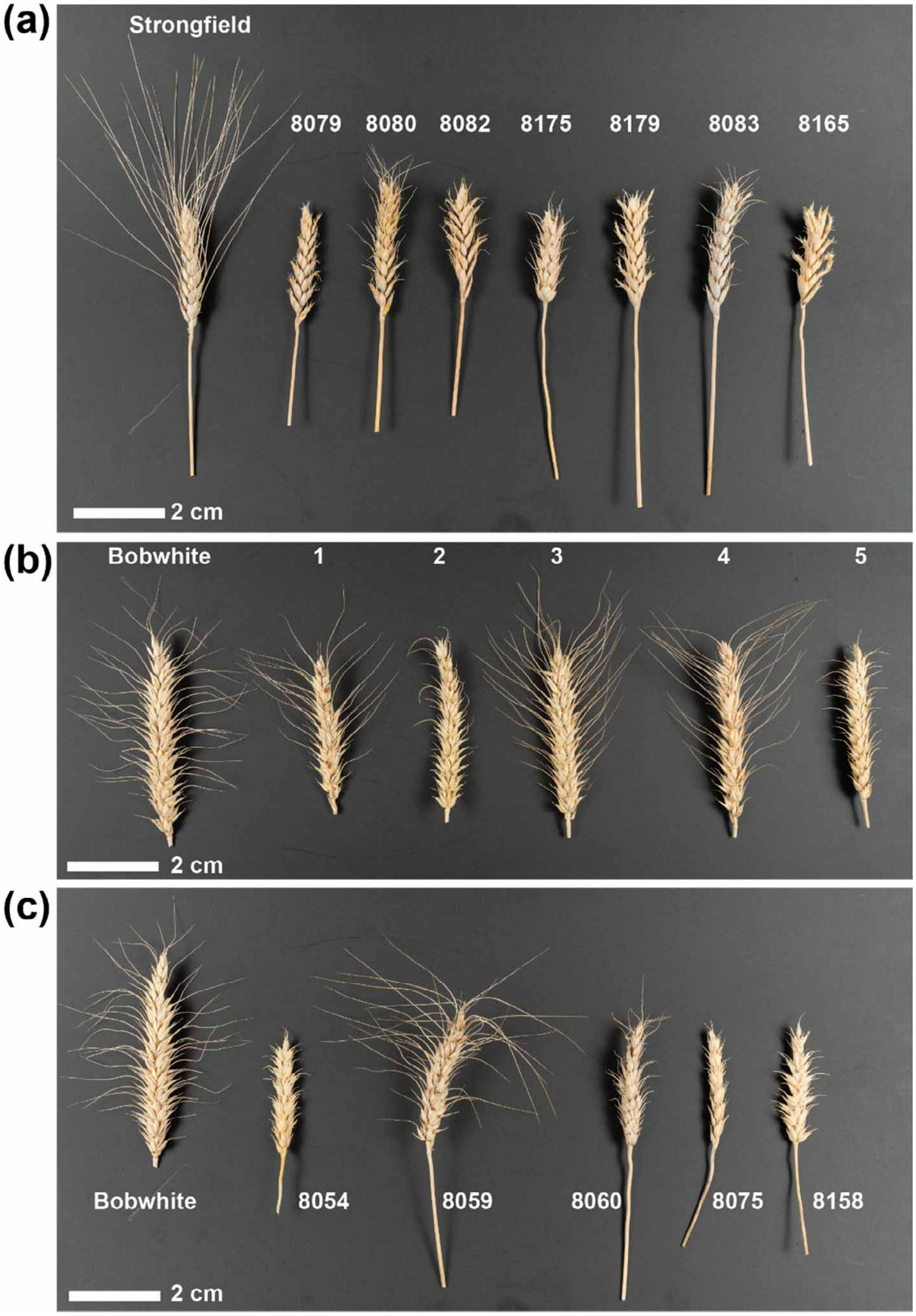

Figure S6. Inflorescence phenotypes of *B1* overexpression lines at maturity.
(a) T_0 lines of the Strongfield durum wheat cultivar compared to wildtype. Line names are denoted in the figure. (b) Segregation of awn phenotypes in T_1 generation of the *B1* overexpression line 7933 from the bread wheat Bobwhite background. Awn lengths were reduced in plants 2 and 5. (c) T_0 generation of *B1* overexpression lines from the Bobwhite cultivar background. Scale bar represents 2 cm.

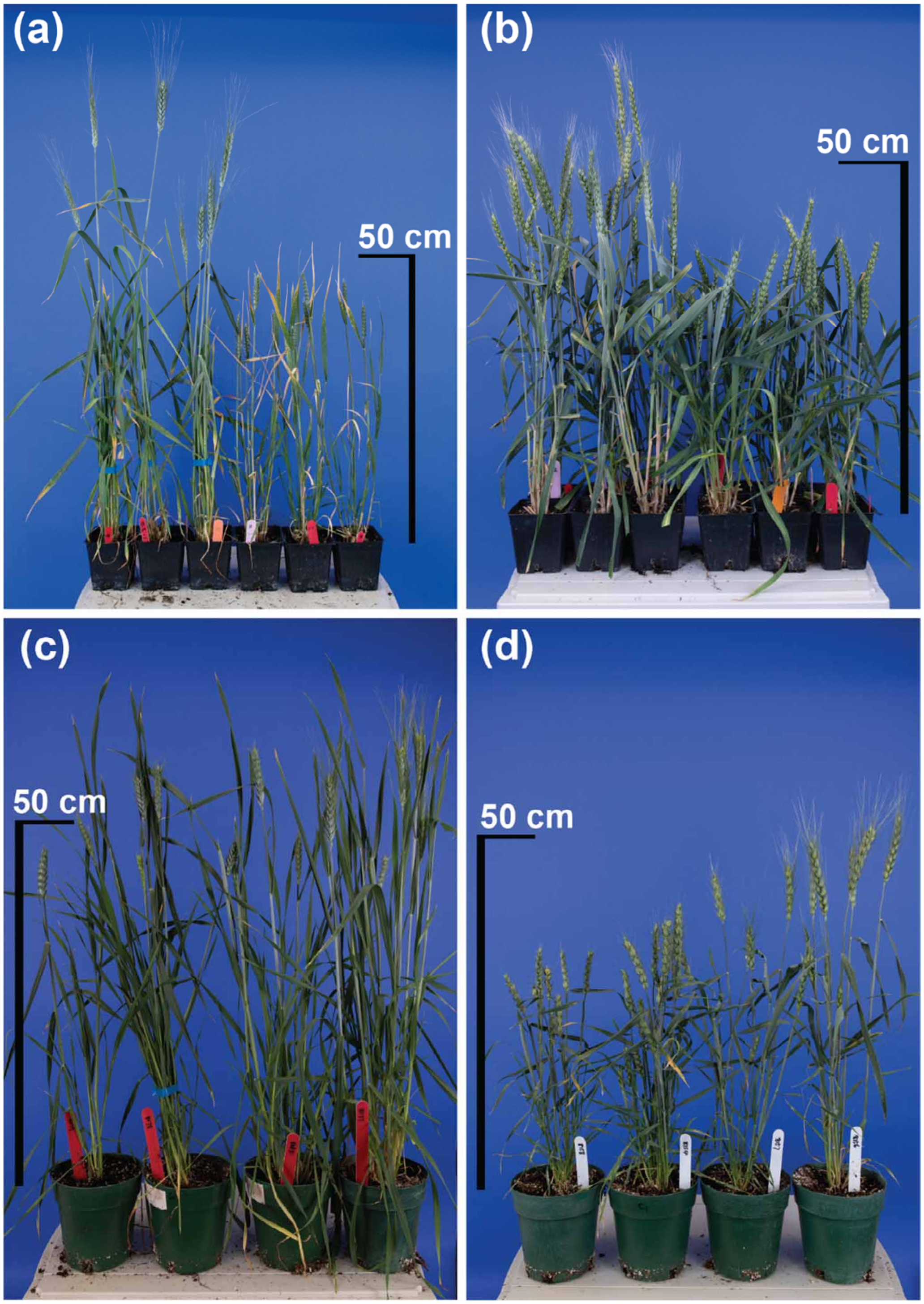

Figure S7. Reduction in plant height and awn length resulting from overexpression of *B1* gene. (a) Segregation of plant height and awn length in the T_1 generation of *B1* overexpression Strongfield line 7888 (durum wheat). (b) Segregation of plant height and awn length in the T_1 generation of *B1* overexpression Bobwhite line 7933 (bread wheat). (c) *B1* overexpression Strongfield T_0 generation lines. (d) *B1* overexpression Bobwhite T_0 generation lines. Scale bars represent 50 cm.

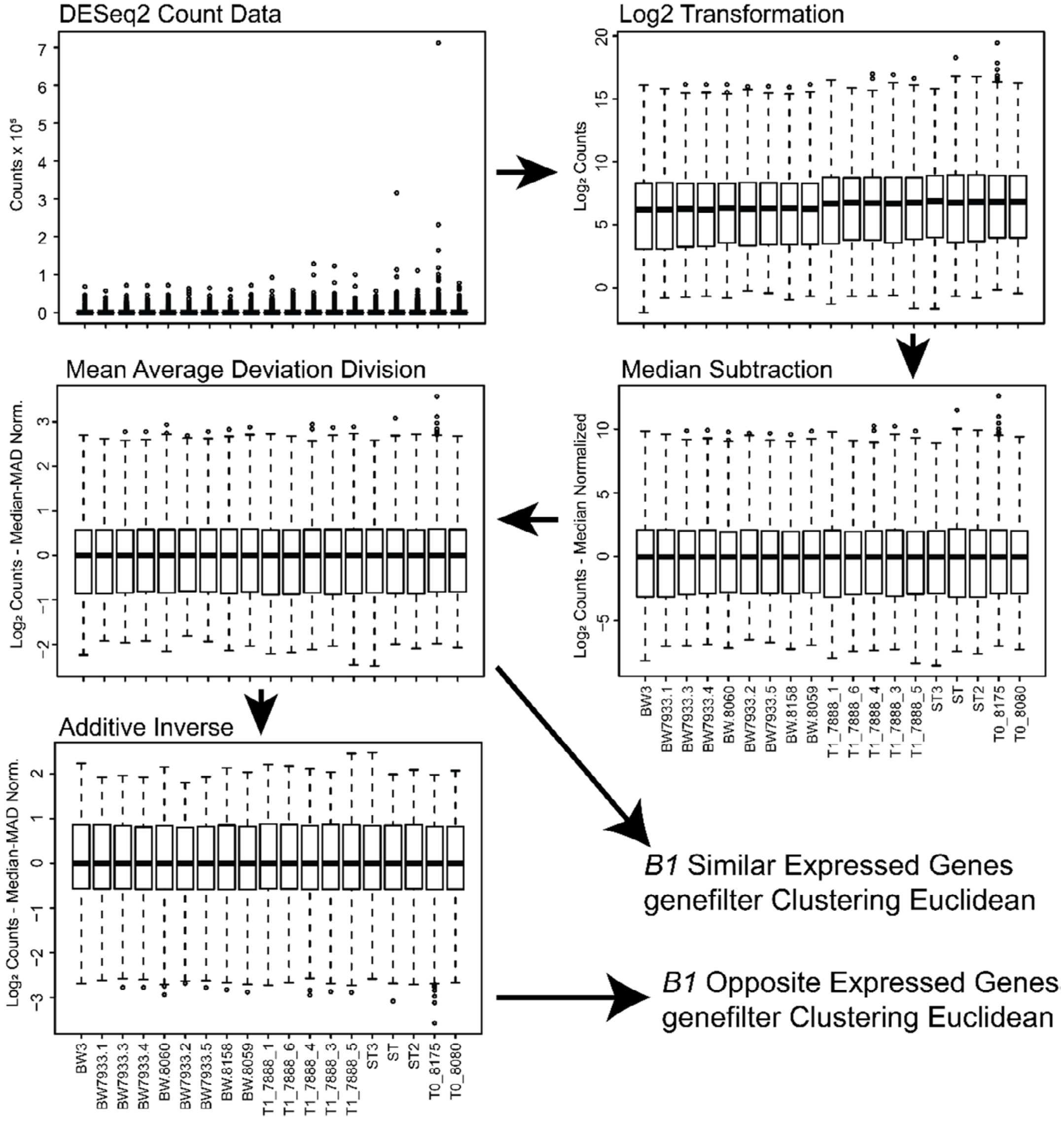

Figure S8. Workflow to identify top one-hundred *B1* similarly and oppositely expressed genes in *B1* overexpression lines.

Data was transformed to log2 and normalized by subtracting the median and dividing by the mean average deviation (median-MAD) of each sample. The genefilter R package was used to find the top 100 similarly expressed genes, to *TraesCS5A02G542800*, based on Euclidean distance. To find genes oppositely expressed to *B1*, the same procedure as finding similarly expressed genes was implemented except that the additive inverse of the median-MAD normalized data was used before proceeding to the genefilter R package (Gentleman *et al.*, 2018).

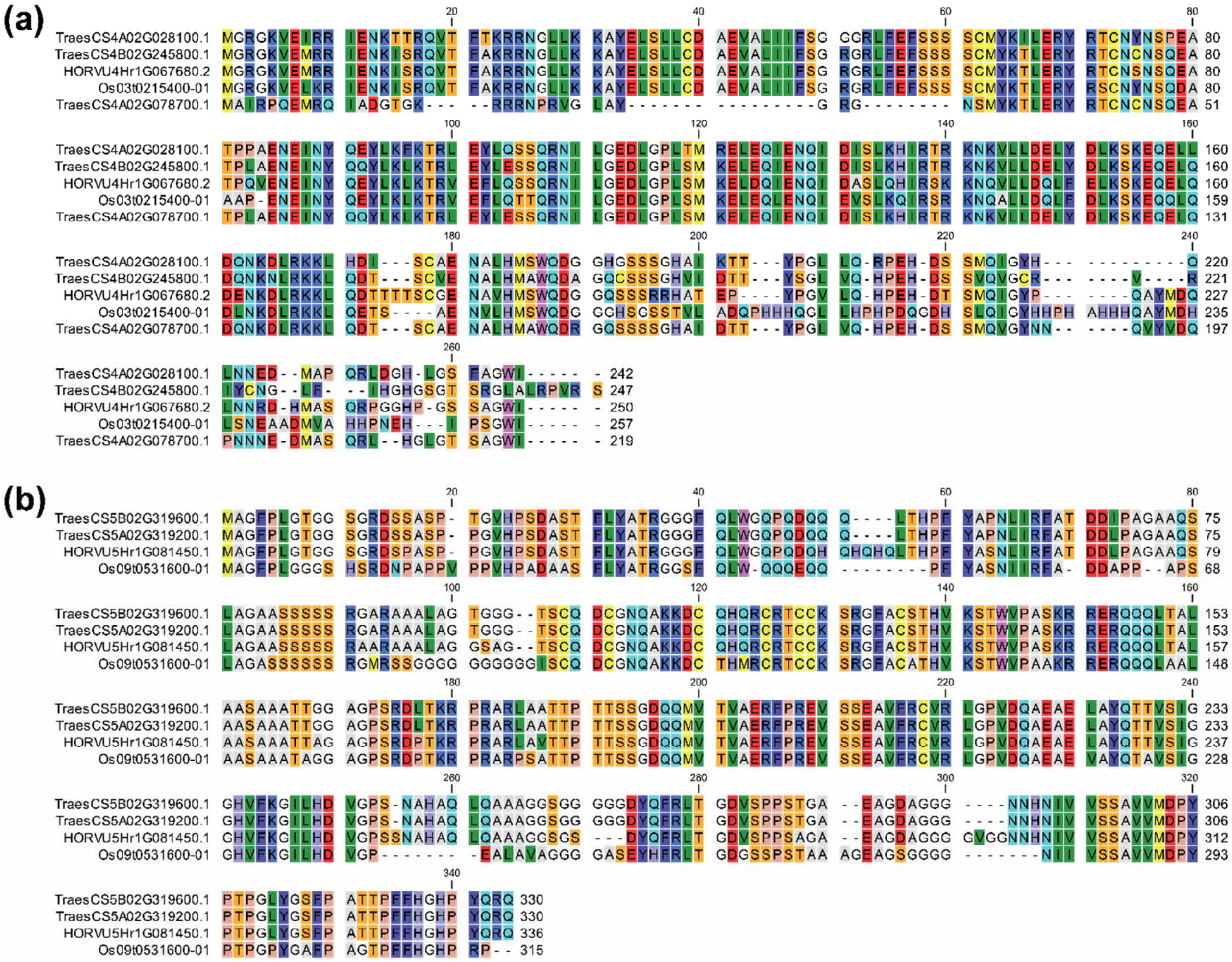

Figure S9, Continued Below. Amino acid alignments of wheat proteins with orthology to MADS1, VRS2, ARF19, and Lks2, whose encoding genes were differentially regulated in *B1* overexpression lines.

(a) Alignment of rice OsMADS1 (Os03t0215400-01), barley MADS-box7 (HORVU4Hr1G067680.2), and wheat proteins TraesCS4A02G078700.1, TraesCS4B02G245800.1, and TraesCS4A02G028100.1. (b) Alignment of barley Six-Rowed Spike 2 (VRS2), HORVU5Hr1G081450.1, with rice Os09t0531600-01 and wheat proteins TraesCS5A02G319200.1 and TraesCS5B02G319600.1. (c) Alignment of barley Short Awn 2 (Lks2), HORVU7Hr1G095030.4, with rice Os06t0712600-00 and wheat protein TraesCS7A02G406300. (d) Alignment of rice ARF19, Os06t0702600-01, to barley HORVU7Hr1G096460.1, maize ZM00001d014690_T006, and wheat protein TraesCS7B2G316600.1. Chinese Spring RefSeq 1.1 proteins shown are translated from the longest predicted splice variant of the transcript.

(c)

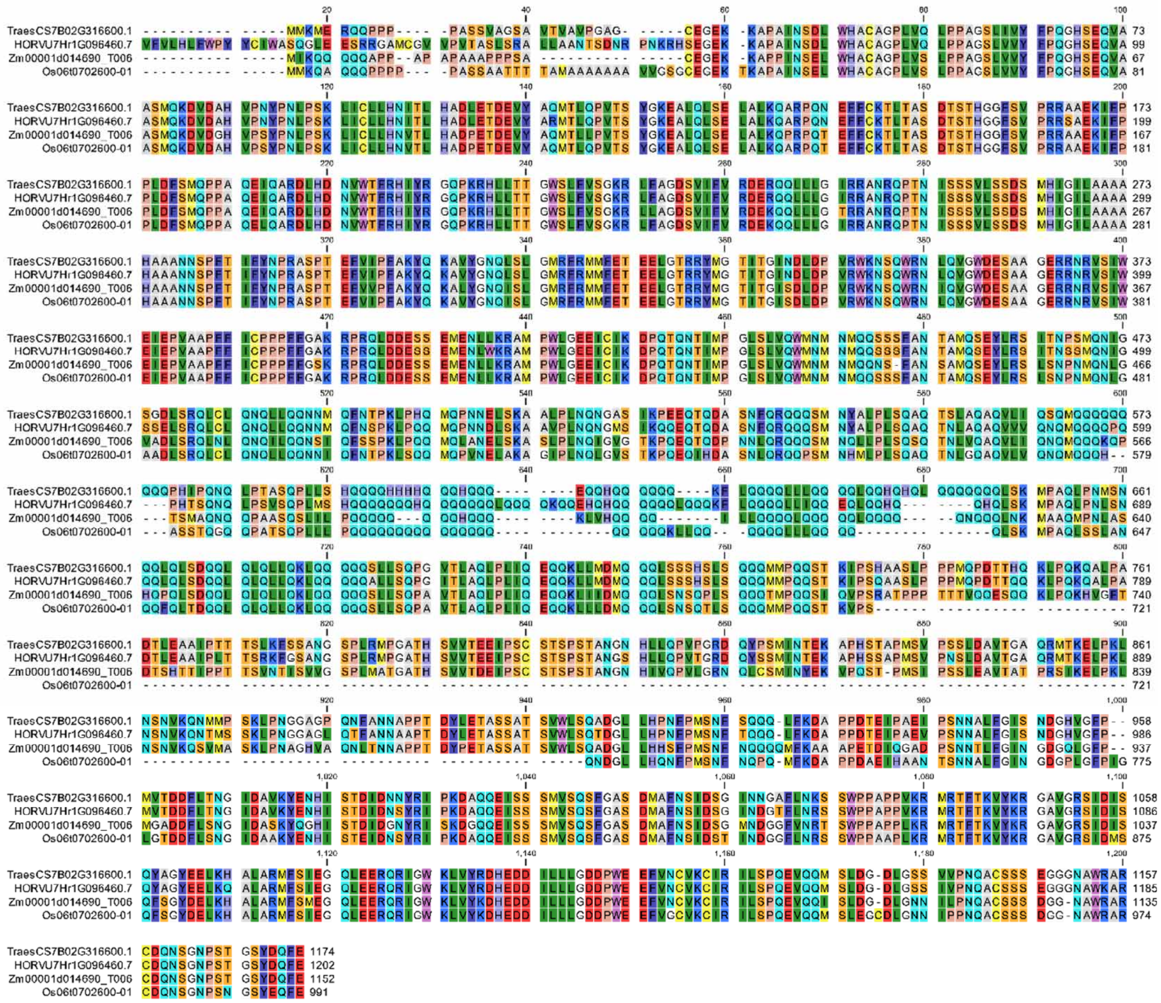

(d)

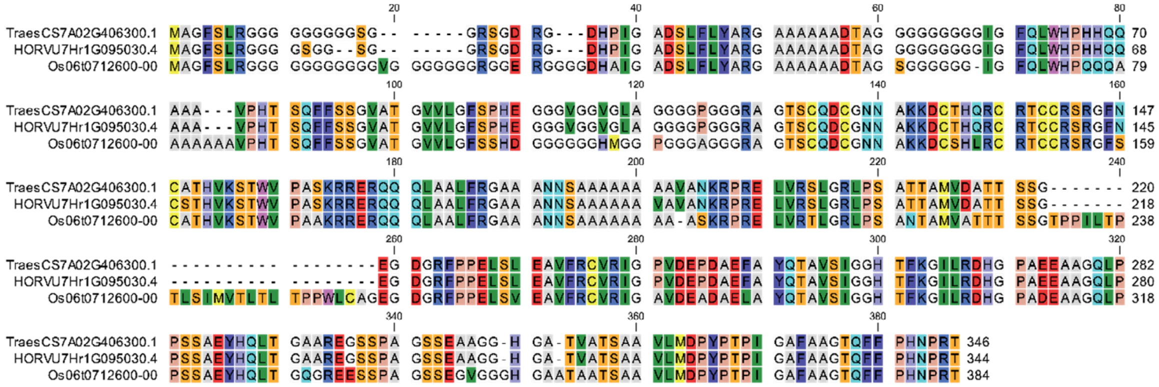

Figure S9. Amino acid alignments of wheat proteins with orthology to MADS1, VRS2, ARF19, and Lks2, whose encoding genes were differentially regulated in *B1* overexpression lines.
See previous page for description.

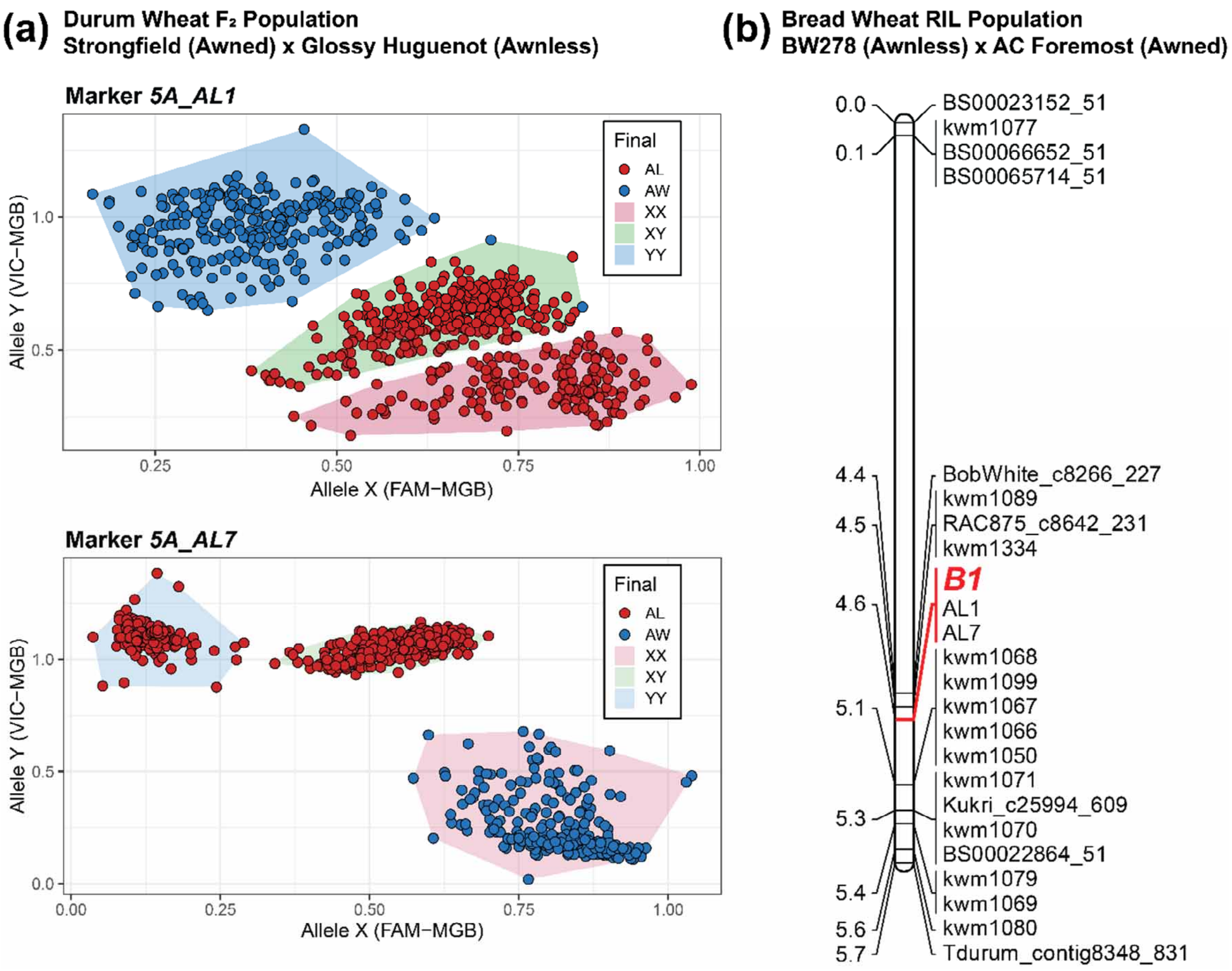

Figure S10. Association of KASP markers *5A_AL7* and *5A_AL1* to awn inhibition.
(a) Genotypes and phenotypes of 800 lines of GH x ST F_2 durum wheat population indicating *5A_AL7* and *5A_AL7* are diagnostic for awned/awnletted trait in this population with 99.9% and 99.4% agreement between genotype and phenotype. AL and AW represent awnletted and awned, respectively. Genotypes are depicted by XX and YY (homozygous) and XY (heterozygous). (b) Fine-mapping of awn trait in a set of 192 lines from BW278 x AC Foremost bread wheat RIL population with *5A_AL7* and *5A_AL1* as well as 21 additional KASP markers between 690,548,798 and 700,443,596-nt on Chinese Spring RefSeq v1.0 revealed that markers *5A_AL7* and *5A_AL1* centered at the *B1* locus. Marker *5A_AL7* is upstream of *B1*, 469-nt from start codon and marker *5A_AL1* is downstream of *B1* at 608-nt from start codon.

SI References

Alexa A, Rahnenfuhrer J. 2018. topGO: Enrichment analysis for gene ontology. R package version 2.34.0. [WWW document] URL https://bioconductor.org/packages/topGO/ [accessed 31 March 2019].

Arabidopsis Genome Initiative. 2000. Analysis of the genome sequence of the flowering plant *Arabidopsis thaliana*. *Nature* **408**: 796-815. doi: 10.1038/35048692.

Avni R, Nave M, Barad O, Baruch K, Twardziok SO, Gundlach H, Hale I, Mascher M, Spannagl M, Wiebe K et al. 2017. Wild emmer genome architecture and diversity elucidate wheat evolution and domestication. *Science* **357**: 93-97. doi: 10.1126/science.aan0032.

Bolger AM, Lohse M, Usadel B. 2014. Trimmomatic: a flexible trimmer for Illumina sequence data. *Bioinformatics* **30**: 2114–2120. doi: 10.1093/bioinformatics/btu170.

Bray N, Pimentel H, Melsted P, Pachter L. 2016. Near-optimal probabilistic RNA-seq quantification. *Nature Biotechnology* **34**: 525-527. doi: 10.1038/nbt.3519.

Broman KW, Gatti DM, Simecek P, Furlotte NA, Prins P, Sen Ś, Yandell BS, Churchill GA. 2019. R/qtl2: Software for mapping quantitative trait loci with high-dimensional data and multiparent populations. *Genetics* **211**:495-502. doi: 10.1534/genetics.118.301595.

Carlson M, Pagès H. 2018. AnnotationForge: Code for building annotation database packages. R package version 1.24.0. [WWW document] URL https://bioconductor.org/packages/AnnotationForge/ [accessed 31 March 2019].

Chen H, Boutros PC. 2011. VennDiagram: a package for the generation of highly-customizable Venn and Euler diagrams in R. *BMC Bioinformatics* **12**: 35. doi: 10.1186/1471-2105-12-35.

Clavijo BJ, Accinelli GG, Wright J, Heavens D, Barr K, Yanes L, Di-Palma F. 2017a. W2RAP: a pipeline for high quality, robust assemblies of large complex genomes from short read data. *bioRxiv.* doi: 10.1101/110999.

Clavijo BJ, Venturini L, Schudoma C, Accinelli GG, Kaithakottil G, Wright J, Borrill P, Kettleborough G, Heavens D, Chapman H et al. 2017b. An improved assembly and annotation of the allohexaploid wheat genome identifies complete families of agronomic genes and provides genomic evidence for chromosomal translocations. *Genome Research* **27**: 885-896. doi: 10.1101/gr.217117.116.

Clavijo B et al. 2019. Sequencing the wheat genome. 10+ Genome Project. [WWW document] URL https://opendata.earlham.ac.uk/opendata/data/ [accessed 31 March 2019].

Durinck S, Spellman PT, Birney E, Huber W. 2009. Mapping identifiers for the integration of genomic datasets with the R/Bioconductor package biomaRt. *Nature Protocols* 4(8): 1184-1191. doi: 10.1038/nprot.2009.97.

Felsenstein J. 1981. Evolutionary trees from DNA sequences: a maximum likelihood approach. *Journal of Molecular Evolution* **17**: 368-376. doi: 10.1007/BF01734359.

Gentleman R, Biocore. 2018. geneplotter: Graphics related functions for Bioconductor. R package version 1.60.0. [WWW document] URL https://bioconductor.org/packages/geneplotter/ [accessed 31 March 2019].

Gentleman R, Carey V, Huber W, Hahne F. 2018. Genefilter: Methods for filtering genes from high-throughput experiments, R package version 1.64.0. [WWW document] URL https://bioconductor.org/packages/genefilter/ [accessed 17 February 2019].

Gu Z, Gu L, Eils R, Schlesner M, Brors B. 2014. circlize Implements and enhances circular visualization in R. *Bioinformatics* **30**: 2811-2812. doi: 10.1093/bioinformatics/btu393

Huber W, Carey VJ, Gentleman R, Anders S, Carlson M, Carvalho BS, Bravo HC, Davis S, Gatto L, Girke T et al. 2015. Orchestrating high-throughput genomic analysis with Bioconductor. *Nature Methods* **12**:115-121. doi: 10.1038/nmeth.3252.

Huang D, Feurtado JA, Smith MA, Flatman LK, Koh C, Cutler AJ. 2017. Long noncoding miRNA gene represses wheat β-diketone waxes. *Proceedings of the National Academy of Sciences USA* **114**: E3149-E3158. doi: 10.1073/pnas.1617483114.

International Brachypodium Initiative. 2010. Genome sequencing and analysis of the model grass *Brachypodium distachyon*. *Nature* **463**: 763-768. doi: 10.1038/nature08747.

International Rice Genome Sequencing Project. 2005. The map-based sequence of the rice genome. *Nature* **436**: 793-800. doi: 10.1038/nature03895.

International Wheat Genome Sequencing Consortium (IWGSC), Appels R, Eversole K, Stein N, Feuillet C, Keller B, Rogers J, Pozniak CJ, Choulet F, Distelfeld A et al. 2018. Shifting the limits in wheat research

and breeding using a fully annotated reference genome. *Science* **361**: eaar7191. doi: 10.1126/science.aar7191.

Jones DT, Taylor WR, Thornton JM. 1992. The rapid generation of mutation data matrices from protein sequences. *Computer Applications in the Biosciences* **8:** 275-282. doi: 10.1093/bioinformatics/8.3.275.

Kersey PJ, Allen JE, Allot A, Barba M, Boddu S, Bolt BJ, Carvalho-Silva D, Christensen M, Davis P, Grabmueller C et al. 2018. Ensembl Genomes 2018: an integrated omics infrastructure for non-vertebrate species. *Nucleic Acids Research* **46(D1)**: D802-D808. doi: 10.1093/nar/gkx1011.

Kinsella RJ, Kähäri A, Haider S, Zamora J, Proctor G, Spudich G, Almeida-King J, Staines D, Derwent P, Kerhornou A et al. 2011. Ensembl BioMarts: A hub for data retrieval across taxonomic space. *Database (Oxford)* **2011**: bar030. doi: 10.1093/database/bar030.

Klaus B, Huber W. 2016. Analysis of RNA-Seq data: gene-level exploratory analysis and differential expression. 11. Gene Ontology Enrichment Analysis. 12. Running topGO. [WWW document] URL https://www.huber.embl.de/users/klaus/Teaching/DESeq2Predoc2014.html [accessed 12 February 2019].

Kolde R. 2018. pheatmap: Pretty heatmaps. R package version 1.0.10. [WWW document] URL https://CRAN.R-project.org/package=pheatmap [accessed 31 March 2019].

Ling HQ, Zhao S, Liu D, Wang J, Sun H, Zhang C, Fan H, Li D, Dong L, Tao Y et al. 2013. Draft genome of the wheat A-genome progenitor *Triticum urartu*. *Nature* **496**: 87-90. doi: 10.1038/nature11997.

Liu S, Yeh CT, Tang HM, Nettleton D, Schnable PS. 2012. Gene mapping via bulked segregant RNA-Seq (BSR-Seq). *PLoS One* **7**: e36406. doi: 10.1371/journal.pone.0036406.

Livak KJ, Schmittgen TD. 2001. Analysis of relative gene expression data using real-time quantitative PCR and the 2(-Delta Delta C(T)) method. *Methods* **25**: 402-408. doi: 10.1006/meth.2001.1262.

Lorieux M. 2012. MapDisto: Fast and efficient computation of genetic linkage maps. *Molecular Breeding* **30**:1231-1235. doi: 10.1007/s11032-012-9706-y.

Love MI, Huber W, Anders S. 2014. Moderated estimation of fold change and dispersion for RNA-seq data with DESeq2. *Genome Biology* **15**: 550. doi: 10.1186/s13059-014-0550-8

Luo MC, Gu YQ, Puiu D, Wang H, Twardziok SO, Deal KR, Huo N, Zhu T, Wang L, Wang Y et al. 2017. Genome sequence of the progenitor of the wheat D genome *Aegilops tauschii*. *Nature* **551**: 498-502. doi: 10.1038/nature24486.

Mackay IJ, Bansept-Basler P, Barber T, Bentley AR, Cockram J, Gosman N, Greenland AJ, Horsnell R, Howells R, O'Sullivan DM et al. 2014. An eight-parent multiparent advanced generation inter-cross population for winter-sown wheat: creation, properties, and validation. *G3 (Bethesda)* **4**: 1603-1610. doi: 10.1534/g3.114.012963.

Mann DG, Lafayette PR, Abercrombie LL, King ZR, Mazarei M, Halter MC, Poovaiah CR, Baxter H, Shen H, Dixon RA et al. 2012. Gateway-compatible vectors for high-throughput gene functional analysis in switchgrass (*Panicum virgatum* L.) and other monocot species. *Plant Biotechnology Journal* **10**: 226-236. doi: 10.1111/j.1467-7652.2011.00658.x.

Mascher M, Gundlach H, Himmelbach A, Beier S, Twardziok SO, Wicker T, Radchuk V, Dockter C, Hedley PE, Russell J et al. 2017. A chromosome conformation capture ordered sequence of the barley genome. *Nature* **544**: 427-433. doi: 10.1038/nature22043.

Maydup ML, Antonietta M, Guiamet JJ, Graciano C, López JR, Tambussi EA. 2010. The contribution of ear photosynthesis to grain filling in bread wheat (*Triticum aestivum* L.). *Field Crops Research* **119:** 48-58. doi: 10.1016/j.fcr.2010.06.014.

Meng L, Li H, Zhang L, Wang J. 2015. QTL IciMapping: Integrated software for genetic linkage map construction and quantitative trait locus mapping in biparental populations. *The Crop Journal* **3**: 269–83. doi: 10.1016/j.cj.2015.01.001.

Ou J, Wang Y, Zhu LJ. 2019. trackViewer: A R/Bioconductor package for drawing elegant interactive tracks or lollipop plot to facilitate integrated analysis of multi-omics data. R package version 1.18.3. [WWW document] URL https://bioconductor.org/packages/trackViewer/ [accessed 31 March 2019].

Paradis E. 2010. pegas: An R package for population genetics with an integrated-modular approach. *Bioinformatics* 26: 419-420. doi: 10.1093/bioinformatics/btp696.

Paterson AH, Bowers JE, Bruggmann R, Dubchak I, Grimwood J, Gundlach H, Haberer G, Hellsten U, Mitros T, Poliakov A et al. 2009. The *Sorghum bicolor* genome and the diversification of grasses. *Nature* 457: 551-556. doi: 10.1038/nature07723.

Ramirez-Gonzalez RH, Uauy C, Caccamo M. 2015. PolyMarker: A fast polyploid primer design pipeline. *Bioinformatics* **31**: 2038-2039. doi: 10.1093/bioinformatics/btv069.

Ramírez-González RH, Borrill P, Lang D, Harrington SA, Brinton J, Venturini L, Davey M, Jacobs J, van Ex F, Pasha A et al. 2018. The transcriptional landscape of polyploid wheat. *Science* **361:** eaar6089. doi: 10.1126/science.aar6089.

R Core Team. 2018. R: A language and environment for statistical computing R Foundation for Statistical Computing, Vienna, Austria. V3.5.1. [WWW document] URL https://www.r-project.org/ [accessed 31 March 2019].

RStudio Team. 2018. RStudio: Integrated development for R. RStudio, Inc, Boston, MA. [WWW document] URL https://www.rstudio.com/ [accessed 31 March 2019].

Schnable PS, Ware D, Fulton RS, Stein JC, Wei F, Pasternak S, Liang C, Zhang J, Fulton L, Graves TA et al. 2012. The B73 maize genome: complexity, diversity, and dynamics. *Science* 326: 1112-1115. doi: 10.1126/science.1178534.

Soneson C, Love MI, Robinson MD. 2015. Differential analyses for RNA-seq: transcript-level estimates improve gene-level inferences. *F1000Res* **4**:1521. doi: 10.12688/f1000research.7563.2.

Stein JC, Yu Y, Copetti D, Zwickl DJ, Zhang L, Zhang C, Chougule K, Gao D, Iwata A, Goicoechea JL et al. 2018. Genomes of 13 domesticated and wild rice relatives highlight genetic conservation, turnover and innovation across the genus *Oryza*. *Nature Genetics* 50: 285-296. doi: 10.1038/s41588-018-0040-0.

Thompson JD, Higgins DG, Gibson TJ. 1994. CLUSTAL W: improving the sensitivity of progressive multiple sequence alignment through sequence weighting, position-specific gap penalties and weight matrix choice. *Nucleic Acids Research* **22**:4673-4680. doi: 10.1093/nar/22.22.4673.

Wang J, Lin M, Crenshaw A, Hutchinson A, Hicks B, Yeager M, Berndt S, Huang WY, Hayes RB, Chanock SJ et al. 2009. High-throughput single nucleotide polymorphism genotyping using nanofluidic dynamic arrays. *BMC Genomics* **10**: 561. doi: 10.1186/1471-2164-10-561

Wickham H. 2011. The split-apply-combine strategy for data analysis. *Journal of Statistical Software* **40**: 1-29. doi: 10.18637/jss.v040.i01.

Wickham H. 2016. ggplot2: Elegant graphics for data analysis. New York, USA: Springer-Verlag.

Wickham H, François R, Henry L, Müller K. 2018a. dplyr: A grammar of data manipulation. R package version 0.7.8. [WWW document] URL https://CRAN.R-project.org/package=dplyr [accessed 31 March 2019].

Wickham H, Henry L. 2018. tidyr: Easily tidy data with 'spread()' and 'gather()' Functions. R package version 0.8.2. [WWW document] URL https://CRAN.R-project.org/package=tidyr [accessed 31 March 2019].

Wickham H, Hester J, Francois R. 2018b. readr: Read rectangular text data. R package version 1.3.1. [WWW document] URL https://CRAN.R-project.org/package=readr [accessed 31 March 2019].

Yu J, Hu S, Wang J, Wong GK, Li S, Liu B, Deng Y, Dai L, Zhou Y, Zhang X et al. 2002. A draft sequence of the rice genome (*Oryza sativa* L. ssp. indica). *Science* 296: 79-92.
